# Genetic deconvolution of fetal and maternal cell-free DNA in maternal plasma enables next-generation non-invasive prenatal screening

**DOI:** 10.1038/s41421-022-00457-4

**Published:** 2022-10-13

**Authors:** Chenming Xu, Jianli Li, Songchang Chen, Xiaoqiang Cai, Ruilin Jing, Xiaomei Qin, Dong Pan, Xin Zhao, Dongyang Ma, Xiufeng Xu, Xiaojun Liu, Can Wang, Bingxin Yang, Lanlan Zhang, Shuyuan Li, Yiyao Chen, Nina Pan, Ping Tang, Jieping Song, Nian Liu, Chen Zhang, Zhiwei Zhang, Xiang Qiu, Weiliang Lu, Chunmei Ying, Xiaotian Li, Congjian Xu, Yanlin Wang, Yanting Wu, He-Feng Huang, Jinglan Zhang

**Affiliations:** 1 Obstetrics and Gynecology Hospital, Institute of Reproduction and Development, Fudan University, Shanghai, China; 2 International Peace Maternity and Child Health Hospital, School of Medicine, Shanghai Jiao Tong University, Shanghai, China; 3 Beijing BioBiggen Technology Co., Ltd, Beijing, China; 4 State Key Laboratory of Genetic Engineering and MOE Engineering Research Center of Gene Technology, School of Life Sciences, Fudan University, Shanghai, China; 5 Jiaxing Maternity and Child Health Care Hospital, Jiaxing, Zhejiang China; 6 Medical Genetics Center, Maternal and Child Health Hospital of Hubei Province, Wuhan, Hubei China; 7 Obstetrics and Gynecology Hospital of Fudan University, Shanghai, China; 8 Shanghai Key Laboratory of Embryo Original Diseases, Shanghai, China; 9 Research Units of Embryo Original Diseases, Chinese Academy of Medical Sciences, Shanghai, China

**Keywords:** Bioinformatics, Comparative genomics

## Abstract

Current non-invasive prenatal screening (NIPS) analyzes circulating fetal cell-free DNA (cfDNA) in maternal peripheral blood for selected aneuploidies or microdeletion/duplication syndromes. Many genetic disorders are refractory to NIPS largely because the maternal genetic material constitutes most of the total cfDNA present in the maternal plasma, which hinders the detection of fetus-specific genetic variants. Here, we developed an innovative sequencing method, termed coordinative allele-aware target enrichment sequencing (COATE-seq), followed by multidimensional genomic analyses of sequencing read depth, allelic fraction, and linked single nucleotide polymorphisms, to accurately separate the fetal genome from the maternal background. Analytical confounders including multiple gestations, maternal copy number variations, and absence of heterozygosity were successfully recognized and precluded for fetal variant analyses. In addition, fetus-specific genomic characteristics, including the cfDNA fragment length, meiotic error origins, meiotic recombination, and recombination breakpoints were identified which reinforced the fetal variant assessment. In 1129 qualified pregnancies tested, 54 fetal aneuploidies, 8 microdeletions/microduplications, and 8 monogenic variants were detected with 100% sensitivity and 99.3% specificity. Using the comprehensive cfDNA genomic analysis tools developed, we found that 60.3% of aneuploidy samples had aberrant meiotic recombination providing important insights into the mechanism underlying meiotic nondisjunctions. Altogether, we show that the genetic deconvolution of the fetal and maternal cfDNA enables thorough and accurate delineation of fetal genome which paves the way for the next-generation prenatal screening of essentially all types of human genetic disorders.

## Introduction

Human birth defects occur in ~3%–5% of liveborns^1^. It has been estimated that 15%–25% of birth defects were attributed to recognizable genetic diseases^2^. There are over 8600 diseases with a known or suspected underlying genetic etiology, most of which have no effective treatments^3^. To provide management options for pregnancies at risk of life-threatening genetic disorders such as sickle cell anemia, Tay-Sachs disease, and cystic fibrosis, population-based genetic screening has been carried out since the 1970s with proven clinical utility^4^. In the past decade, non-invasive prenatal screening (NIPS) was developed and implemented worldwide to screen for common fetal chromosomal aneuploidies such as trisomy 21 (T21, also known as Down syndrome)^5^. Recently, NIPS has expanded to cover chromosomal microdeletion and microduplication syndromes (MMS) such as DiGeorge syndrome^6^. Studies have also demonstrated that fetal cell-free DNA (cfDNA) is useful for the diagnosis or screening of common monogenic conditions such as achondroplasia and Noonan spectrum disorders^7,8^. However, a comprehensive NIPS test for concurrent screening of chromosomal and monogenic disorders has yet to be developed and implemented to battle the large catalog of genetic birth defects.

NIPS utilizes a simple draw of maternal blood containing both maternal and fetal circulating cfDNA^5^. Fetal cfDNA is believed to be derived from the apoptotic cells of the outer layer of placental trophoblast, which then enters maternal circulation^9^. The fetal cfDNA accounts for only a small proportion of the total cfDNA in maternal plasma which is estimated only ~10% on average during the 2nd trimester and even lower during late 1st trimester when NIPS is offered clinically^10^. At such a low level, the fetal variants can only be recognized when any detectable difference introduced by the fetal genetic abnormality (signal) exceeds that associated with assay variations (noise). In previous studies, up to ~11,000–20,000 loci were used in NIPS to detect common aneuploidies^11,12^. Apparently, the single nucleotide polymorphism (SNP)-based NIPS can be improved by increasing the signal-to-noise ratio so that far fewer SNP loci need to be interrogated. Besides the analytical challenge of detecting low-level fetal variants in the maternal and fetal cfDNA admixture, accurate NIPS results are often obscured by multiple gestations, maternal germline copy number variations (CNVs), absence of heterozygosity (AOH), and other analytical challenges^13–15^. In twin pregnancies, the individual cfDNA contributed by each fetus is usually lower than that of a singleton fetus affected with an aneuploidy which exacerbates the NIPS assay sensitivity^16^. In addition, ultrasound may not accurately identify twin zygosity or vanishing twins which can cause increased health risks for the fetus(es). Maternal CNVs such as non-pathogenic duplications cause a significant number of false-positive fetal trisomies detected by NIPS^17^. Maternal AOH reduces the number of the interpretable heterozygous loci for the detection of fetal chromosomal copy number abnormalities in NIPS assays depending on genotype information^18^. These confounders, if not properly recognized, lead to false screening results, which in turn may result in missed diagnoses or unnecessary invasive procedures for diagnostic confirmation.

Currently, there are two prevailing NIPS approaches that involve low-coverage whole genome sequencing (WGS) or targeted sequencing for SNPs to infer fetal chromosome copy number^5,11^. WGS-based NIPS utilizes chromosome-specific read depth (RD) data to identify fetal chromosomal aberrations based on a *Z*-score calculation^5^. SNP-based NIPS detects chromosomal aberrations at selected loci by the quantitation of skewed allelic fraction (AF) caused by CNVs^19^. These two methods have distinct strengths and limitations for the analyses of fetal cfDNA. For instance, SNP-based NIPS is limited in detecting fetal copy number changes at regions with maternal AOH^18,19^. The low-coverage WGS method is constrained due to its inability to discriminate maternal and fetal genotypes, which limits its clinical utility for the detection of hydatidiform moles or unrecognized twin or vanishing twin pregnancies^13^. The combined use of RD and AF for high-coverage NIPS has been proposed from a simulation dataset, but the clinical validity of this approach needs to be substantiated by larger studies^20^.

Besides the RD and AF data mentioned above, the cfDNA fragmentation pattern can also be informative for the detection of fetal variants in NIPS. The size of fetal cfDNA fragment is usually shorter than the maternal counterpart^21,22^. The assay sensitivity can be improved by sampling shorter cfDNA fragments from the maternal plasma when a higher percentage of fetal cfDNA molecules are recovered in the NIPS test^23,24^. Human aneuploidies were reported to be associated with aberrant maternal meiotic recombination presenting with an erratic number of crossovers and unusual chromosome breakpoints^25–27^. Therefore, the discovery of chromosome recombinants would serve as additional evidence for the detection of aneuploidies in NIPS. However, aberrant meiotic crossovers associated with human aneuploidies have not been reported in cfDNA likely due to current assay limitations. Overall, the recognition of fetus-specific cfDNA characteristics including its fragment length or meiotic crossovers can improve the performance of NIPS as additional information is utilized to discriminate the fetal variants from the maternal background.

In this study, we present a new NIPS approach employing non-biased allelic target enrichment followed by next-generation sequencing (NGS) for the comprehensive analyses of fetal chromosomal aneuploidies, MMS, and monogenic disorders. This approach uses deep sequencing to analyze genomic regions associated with three distinct types of genetic disorders with test resolution from single base variant to whole chromosome copy number change. In addition, this method genetically deconvolutes the fetal and maternal cfDNA admixture by querying NGS data associated with maternal/fetal genotype, RD, SNP linkage, and cfDNA fragment lengths. With the concerted analysis of multidimensional genomic cues, this new NIPS assay yielded much improved analytical performance and addressed the limitations of current methods caused by multiple assay confounders. Furthermore, we discovered meiotic errors associated with aberrant chromosome recombinants from the cfDNA studies providing important insights into the origins of human aneuploidies.

## Results

### Coordinative allele-aware target enrichment suppresses allelic hybridization bias

**Fig. 1:**
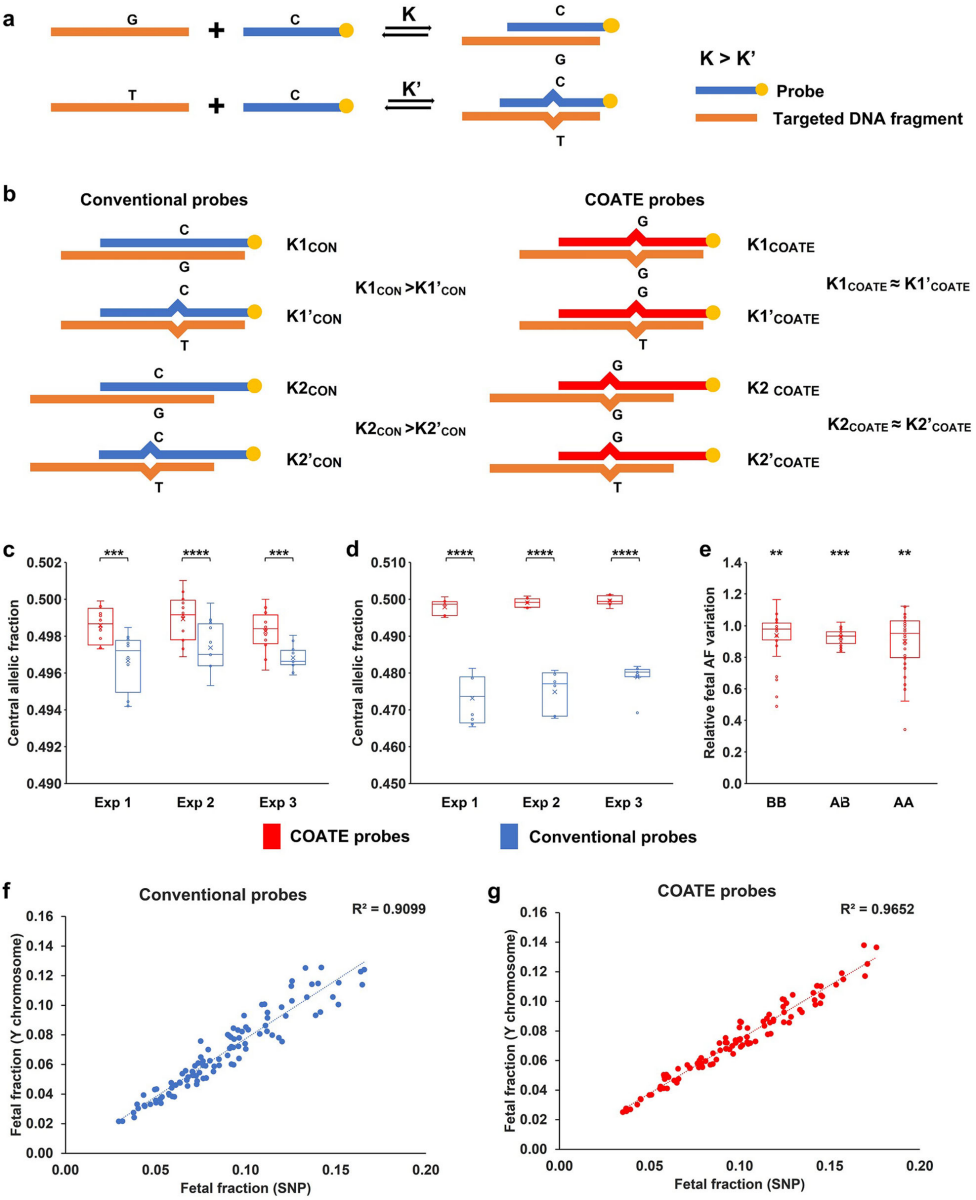
COATE-seq suppresses allelic hybridization bias. **a** Enrichment of targeted genomic region by liquid-phase hybridization. Probe and target strand pairing is a reversible process, characterized by an equilibrium constant. The equilibrium constant for a probe complementary to the target sequence (K) is larger than that for its target sequence with a variant (*K*’). **b** Unlike conventional (CON) probes, COATE probes do not discriminate reference and alternative alleles, and thus the difference between *K*_COATE_ and *K*_COATE_’ is smaller than that of *K*_CON_ and *K*_CON_’. Two representative DNA fragments with different hybridization equilibrium constants were shown for conventional and COATE probes. **c** The CAF (central allelic fraction) in maternal heterozygous loci of 12 pregnant women’s plasma samples was significantly higher and closer to 0.5 when the COATE probes were used at hybridization temperature of 65 °C. The experiment was repeated three times. **d** The reduction in hybridization allelic bias was also significant at hybridization temperature of 68 °C using eight pregnant women’s plasma samples. The experiment was repeated three times. **e** The CV of AFs was compared between COATE-seq and CON-seq (enrichment performed by COATE and conventional probes, respectively) at hybridization temperature of 65 °C. The ratios were shown for the CV using COATE-seq and CON-seq at loci where the mother was homozygous for the reference allele (BB), homozygous for the alternative alleles (AA), and heterozygous (AB). Paired *t*-test was used in **c**–**e** for the comparisons; *P < 0.05, **P < 0.01, ***P < 0.001, and ****P < 0.0001. **f**, **g** The comparison of the FF calculation using the COATE-seq and CON-seq methods. FF was calculated for 102 male pregnancies based on the SNP and canonical Y-chromosome method.

The fetal fraction (FF) is ~10% on average during the 2nd trimester and even lower during late 1st trimester present in maternal plasma^10^. The variation of SNP AF caused by fetal chromosomal copy number abnormality is subtle and only detectable when it exceeds that caused by assay artifacts. In conventional liquid-phase hybridization for target enrichment, oligonucleotide probes are designed to perfectly match reference sequences (Fig. 1a). When the number of complementary base pairs is increased, the total gain in free Gibbs energy-related duplex formation rises^28^. Therefore, DNA fragments harboring the reference allele (wild-type allele) have a higher pairing affinity to probes than those with the alternative allele (variant allele; Fig. 1a, b). This small but significant allelic bias disfavoring the alternative allele confounds the NIPS assay for the detection of low-level fetal chromosome CNV. We found that the average AF at the maternal heterozygous loci was always lower than 0.5, indicating that fewer DNA fragments with variant alleles were recovered than those with the wild-type alleles (Fig. 1c, d; Materials and methods). To reduce such allelic biases, probes were designed for SNPs on different chromosomes associated with the most common human aneuploidies or MMS (see Materials and methods), which have a minimal difference of the hybridization equilibrium constants for the reference and alternative alleles (Δ*K* = *K*– *K*’ ≈ 0; Fig. 1b). Noteworthily, the sequences of these probes may not be perfectly complementary to either the reference or alternative alleles (Fig. 1b; Materials and methods). This new target enrichment strategy for NGS was named coordinative allele-aware target enrichment sequencing (COATE-seq). We found that the COATE-seq yielded less allelic bias for hybridization-based target enrichment than sequencing performed with conventional probes (CON-seq; Fig. 1c–e). The median of the AF across all maternal heterozygous loci, was significantly elevated and closer to 0.5 when COATE-seq was performed (Fig. 1c, d). In addition, the AF coefficient of variation (CV) of fetal variants was also significantly reduced in COATE-seq (Fig. 1e; Supplementary Fig. S1). To evaluate whether COATE-seq was indeed beneficial for fetal SNP AF quantification, the FF determined by the AF values of all informative SNP loci was compared to that calculated by the Y chromosome-based method. The COATE-seq yielded a higher correlation of coefficient (*R*^2^ = 0.97) for the SNP-based method than that of CON-seq (*R*^2^ = 0.91; Fig. 1f, g). Consistently, we found that the COATE-seq yielded a smaller bias for the FF estimation when using the fetal heterozygous SNPs at loci where the mother was homozygous for the reference or alternative allele (Supplementary Fig. S2). Overall, the above results demonstrate that COATE-seq produces more accurate quatification for fetal SNP AF than the conventional enrichment method which is critical to improve the signal-to-noise ratio in the NIPS assay for the detection of chromosomal aberrations.

### Multidimensional cfDNA analyses for the detection of fetal chromosome CNV

**Fig. 2:**
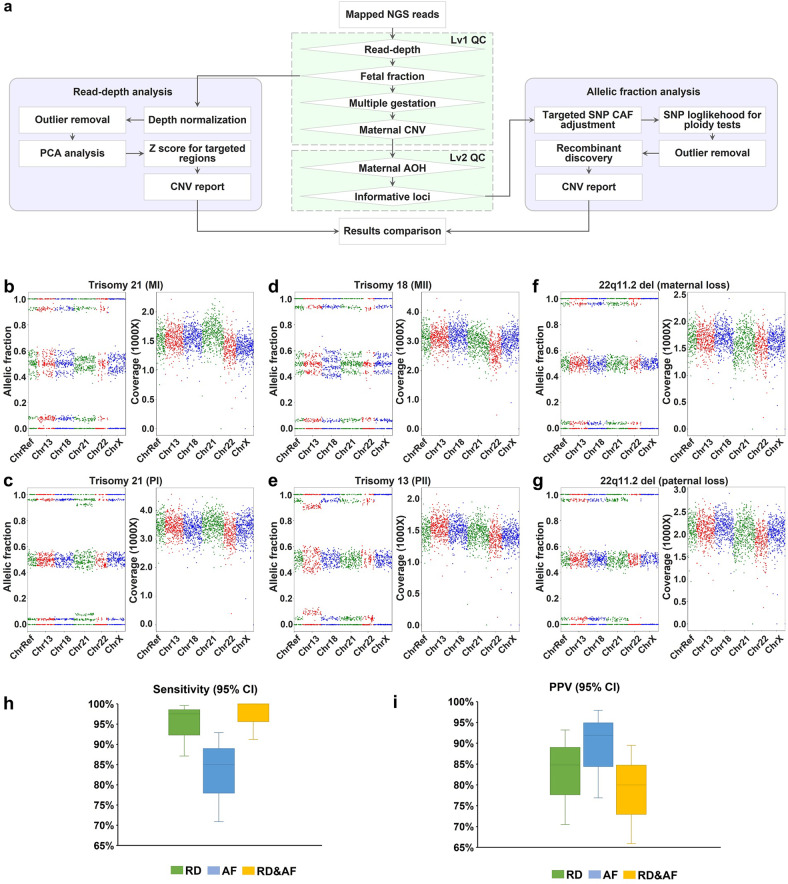
Combined SNP allelic ratio and RD analysis detects fetal chromosomal aberrations. **a** Detection of fetal chromosomal disorders involves a combined analysis of SNP AF and RD. Lv1 QC includes monitoring of sufficient RD, FF, multiple gestations, and maternal CNV. Samples passing Lv1 QC are subjected to the RD-based chromosomal analysis. Lv2 QC includes the detection of maternal AOH and calculation of the number of informative loci where the fetus carries a heterozygous allele, and the mother is homozygous. Samples passing both Lv1 and Lv2 QC undergo the SNP AF-based chromosomal analysis. **b**–**g** Representative results of common aneuploidies and MMS. Each case contains results of SNP AF (left panel) and SNP coverage (right panel). Shown on panel **b**–**g**, chromosome-specific SNP loci are colored in different groups which are spanning mappable regions on chr13, chr18, chr21, and chrX. The SNPs shown for chr22 are those on the critical region associated with DiGeorge syndrome (chr22:17,322,843-21,118,912). The SNPs in the chrRef group include reference SNPs not on chr13, chr18, chr21, chr22, chrX, and chrY (see Materials and methods). A T21 case due to MI NDJ (**b**). A T21 case due to PI NDJ (**c**). A T18 case due to MII NDJ (**d**). A T13 case due to PII NDJ (**e**). A 22q11.2 microdeletion case with a loss of maternal chr22 segment (**f**). A 22q11.2 microdeletion case with a loss of paternal chr22 segment (**g**). **h**, **i** The values of sensitivity (**h**) and PPV (**i**) are shown by different analytic methods. Bars indicate 95%CI. AF allelic fraction, RD read depth, RD&AF combined analysis for RD and AF, CI confidence interval, PPV positive predictive value, MMS microdeletion and microduplication syndrome, SNP single nucleotide polymorphism, PCA principal component analysis, CAF central allelic fraction, QC quality control, Lv1 level 1, Lv2 level 2, FF fetal fraction.

**Fig. 3:**
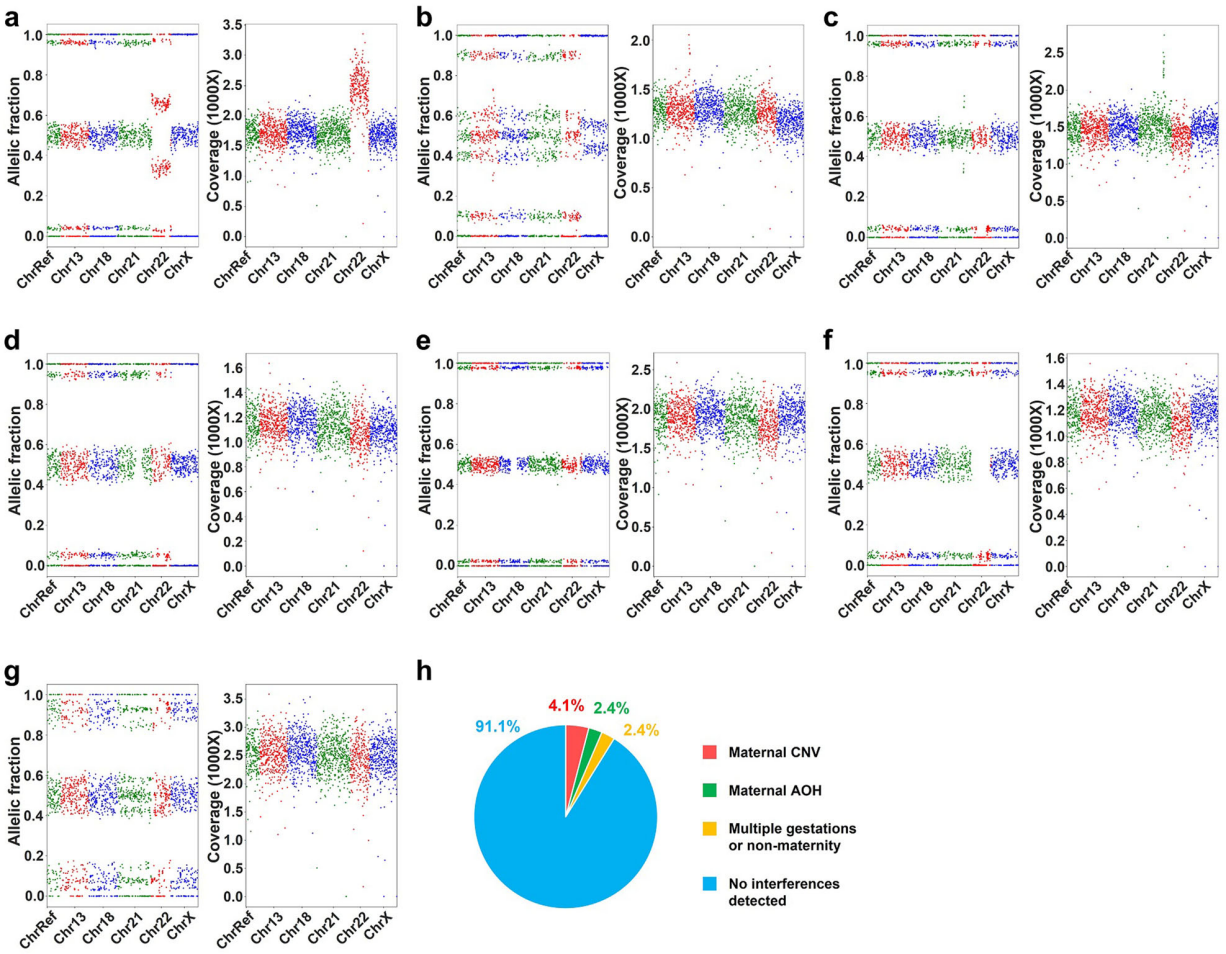
Genetic deconvolution of fetal and maternal cfDNA admixtures precludes maternal interference. **a**–**c** Representative cases with maternal CNV revealed by changes in both the SNP AF and RD panels, including a ~3.1 Mb duplication (**a**), a ~0.35 Mb duplication (**b**), and a ~0.5 Mb duplication (**c**). **d**–**f** Representative cases with maternal AOH revealed by changes in the SNP AF panel only, including a ~3.2 Mb AOH region on chr21 (**d**), a ~16 Mb AOH region on chr18 (**e**), and a ~3.1 Mb AOH region on chr22 (**f**). **g** A representative case of a dizygotic twin pregnancy with both an increased number of detected fetal SNPs and their AF variation. **h** Percentages of cases with multiple gestations or non-maternity, and cases with maternal CNV (size > 200 kb) or maternal AOH regions (≥ 75 consecutive homozygous SNPs) detected on chr13, chr18, chr21, chr22, and chrX. Probes span essentially entire mappable regions on chr13, chr18, chr21, and chrX. The SNPs shown for chr22 are those on the critical region associated with DiGeorge syndrome (chr22:17,322,843-21,118,912, see Materials and methods). CNV copy number variation, SNP single nucleotide polymorphism, AOH absence of heterozygosity, AF allelic fraction, RD read depth.

While COATE-seq improves fetal variant genotyping accuracy, multiple gestations, maternal CNV, and AOH can confound the detection of abnormal fetal variant intermixed in the total maternal plasma cfDNA. Such obstacles can be overcome when the fetal and maternal genetic profiles are separated at large. To this end, we developed algorithms for concurrent analyses of chromosome dosage and genotype by surveying both the RD and AF data as opposed to conventional NIPS tests depending on either RD or AF data only (Fig. 2a–i). Using this joint analysis, dizygotic twin pregnancies and maternal CNV and AOH can be detected prior to fetal variant calling as a quality control step to reduce analytical errors (Fig. 2a). In 419 samples used for analytical validation, there were 17 (4.1%) with maternal CNV with a size of ≥ 200 kb (Fig. 3a–c), 10 (2.4%) with maternal AOH regions with ≥ 75 consecutive homozygous loci (Fig. 3d–f), and 10 (2.4%) with multiple gestations or non-maternity (Fig. 3g, h). After accounting for confounders including ≥ 3 Mb maternal CNV, maternal chromosomal AOH diminishing heterozygous SNP loci, and non-singleton pregnancy, the RD-based analysis identified all aneuploidies but one T18 resulting in a test sensitivity at 97.5% (95% confidence interval (CI), 87.1%–99.6%) and a positive predictive value (PPV) at 84.8% (95% CI, 70.5%–93.2%) (Fig. 2h, i; Supplementary Fig. S3). The AF-based method detected 34 out of 40 aneuploidies producing an 85.0% (95% CI, 70.9%–92.9%) sensitivity and a 91.9% (95% CI, 76.9%–97.9%) PPV (Fig. 2h, i). The combined RD and AF approach detected all aneuploidies with a 100% (95% CI, 91.2%–100%) sensitivity and an 80.0% (95% CI, 65.9%–89.5%) PPV (Fig. 2h, i). These results demonstrated that the combined use of RD and AF data had a higher test sensitivity than either method used alone. Importantly, the AF-based method is useful to calculate FF and recognize multiple gestations and maternal AOH (Figs. 2a, 3d–g). In addition, the AF-based method is highly sensitive for the detection of fetal chromosomal aberrations derived from paternal meiotic errors (Fig. 2c, e, g). Another added advantage of the AF-based method is the ability to detect the parental and meiotic origin of fetal chromosomal aberrations (Fig. 2b–g; Supplementary Fig. S4a–c). On the other hand, RD analysis complements the AF analysis for cases in which the number of informative loci for the detection of CNV is reduced (Supplementary Fig. S4d).

### Characterization of chromosome recombinants and origins of meiotic nondisjunction (NDJ) by fetal cfDNA profiling

**Fig. 4:**
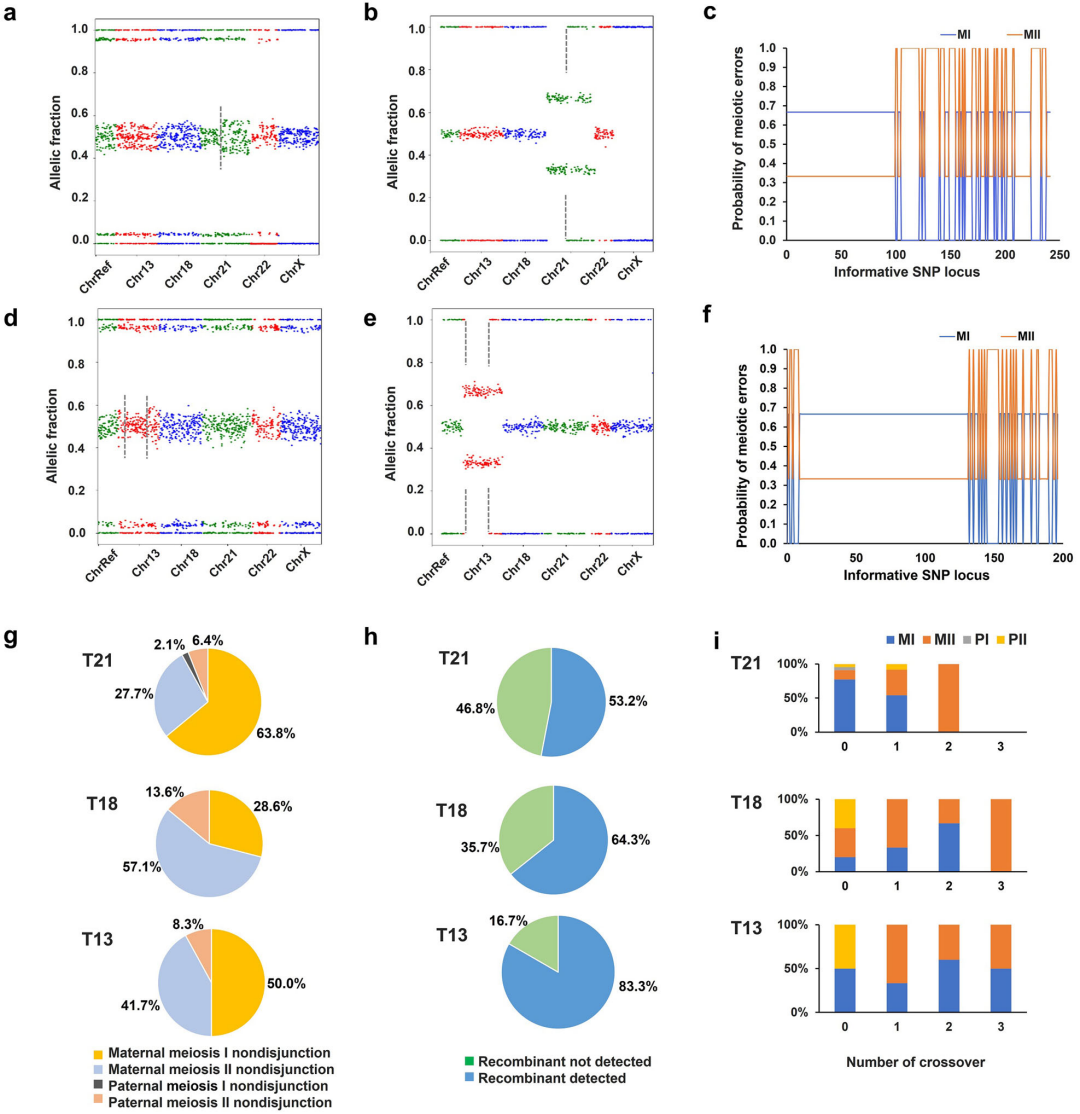
The fetal cfDNA study reveals parental and meiotic origin of NDJ and homologous recombination associated with aneuploidy. **a**–**c** A T21 case using matched maternal and fetal mixed DNA, which shows homologous recombination. The occurrence of recombinant could be inferred from the presence of two different AF patterns consistent with MI and MII NDJs (**a**). In the respective amniocytes, homozygous SNPs (BBB or AAA) are detected only in the telomeric but not centromeric region of chr21 consistent with the presence of recombinant (**b**). At loci where the mother is heterozygous, fetal homozygosity (AAA or BBB) is consistent with MII NDJ. The probability of meiotic errors for each informative locus is plotted (**c**). When fetal genotype is heterozygous (ABB or AAB), the prior probability for the detectable MI NDJ is 2/3 while that for MII NDJ is 1/3 assuming an equal incidence in MI and MII NDJs. The dashed lines indicate the transition of MI and MII SNP patterns suggesting a crossover. **d**–**f** The cfDNA collected from a pregnant woman carrying a T13 fetus shows the recombinant with the respective fetal genomic DNA SNP pattern (**e**) and probability of meiotic errors (**f**). The dashed lines show where transition of MI and MII SNP patterns occurs indicating two crossover events. **g** Percentages of different types of meiotic errors detected. **h** Percentages of aneuploidy cases with and without detectable recombinants. **i** The number of crossovers associated with different types of meiosis NDJ. MI maternal meiosis I, MII maternal meiosis II, PI paternal meiosis I, PII paternal meiosis II, NDJ nondisjunction.

It is known that maternal age is an important risk factor for common aneuploidies, e.g., T21, T18, and T13^29^. These aneuploidies are mostly of maternal origin and associated with aberrant meiotic recombination^25–27^. However, evidence for the interplay between meiotic crossovers and human aneuploidies is scarce likely due to the difficulty of collecting a large number of human eggs or invasively collected prenatal specimens. Given these challenges, the NIPS approach would seem to be an ideal alternative. We then examined whether recombinants associated with aneuploidies could be detected using our COATE-seq method, which had improved analytical performance over conventional approaches (Supplementary Figs. S5, S6; Materials and methods). In 33 sets of samples consisting of matched fetal, maternal DNA, and their admixtures, we identified the chromosome recombinants in mixed DNA and confirmed the recombinants in the fetal genome based on linked SNPs (Fig. 4a–c; Materials and methods). Similarly, the homologous chromosome recombinants can be detected using the same method for the plasma cfDNA samples from pregnant women and their respective amniocytes (Fig. 4d–f; Supplementary Fig. S7). In 73 aneuploidy samples tested in this study, 47 T21 (63.8%) cases had maternal meiosis I (MI) NDJ, with the remaining in maternal meiosis II (MII) NDJ (27.7%), paternal meiosis I (PI) NDJ (2.1%), and paternal meiosis II (PII) NDJ (6.4%), respectively (Fig. 4g; Supplementary Table S1). While MI NDJ (50.0%) was more frequent than other meiotic errors in T13 as seen in T21, MII NDJ (57.1%) was the most frequent one in T18 (Fig. 4g; Supplementary Table S1). Among these samples, 44/73 (60.3%; 25 T21, 9 T18, and 10 T13) with recombinants were detected (Fig. 4h; Supplementary Table S1 and Fig. S8). In the validation set, 34/40 (85.0%) aneuploidies were identified by the AF method when recombination was considered (Fig. 2h), but the detection rate dropped to 67.5% if crossovers were disregarded. Next, we characterized the crossovers associated with the origins of meiotic errors. Altogether, we found that T21 cases with detectable recombinants had more with a single crossover in MI, while in T18 and T13, more cases had two or more crossovers (Supplementary Table S1). The distribution of meiotic errors and crossovers identified in this cohort was consistent with previous reports (Fig. 4h, i)^25–27^. For instance, in T21 due to maternal NDJ with a single crossover, most MI cases had breakpoints near the telomeric region, while breakpoints near the centromeric region were more frequent in MII cases (Supplementary Fig. S9). Again, these results were consistent with previous studies characterizing the occurrence of meiotic recombination in T21 using invasive fetal samples^30^. Overall, these data demonstrated that our NIPS method was highly accurate in characterizing the origin of meiotic errors and crossovers in common aneuploidies.

### Improved detection of fetal de novo and paternally inherited monogenic variants based on fetal cfDNA fragment characteristics

**Fig. 5:**
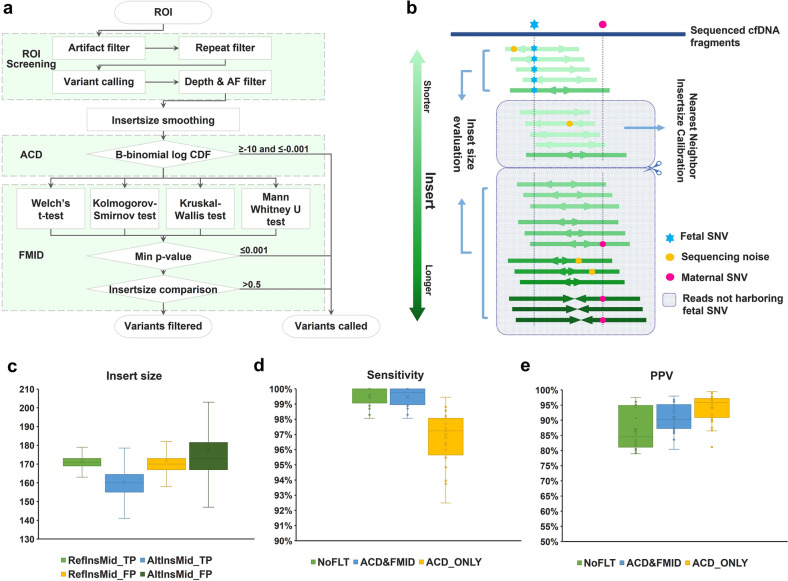
The use of cfDNA characteristics for the detection of fetal monogenic variants. **a** The identification of fetal monogenic variants includes ROI analysis, ACD filtering, and FMID filtering. **b** Fetal-Maternal Nearest Neighbor Insert-size Calibration was first used to exclude those reads harboring wild-type alleles which possess the closest cfDNA fragment length to the reads harboring the variant alleles of a potential fetal origin. The remaining fragments with the wild-type allele were compared with those with the variant alleles for their lengths to identify potential fetal SNVs. **c** Different insert-size distribution for wild-type (ref) and variant (alt) allele supporting reads on 28 samples tested. For all variants detected, median insert-size of ref (RefinsMid) and alt (AltinsMid) allele supporting reads were box plotted with upper whisker (Q3 + 1.5 × IQR), Q3, Q2, average, Q1, and lower whisker (Q1 – 1.5 × IQR) to demonstrate the differences between the TP or FP variants. Insert-size was ~10 bp shorter (*P* < 1.0 × 10^−15^, two-tailed unequal-variance *t*-test) in alt allele group compared to ref allele group on TP variants consistent with a fetal origin and no such difference was seen in FP variants. **d**, **e** Sensitivity (**d**) and PPV (**e**) comparison for different filtering methods using 28 validation samples. By applying both the ACD and FMID variant filters, the test sensitivity was essentially unchanged at 99.5% while the PPV was significantly improved (*P* < 0.01). When only the ACD filter was used, the test sensitivity was reduced to 96.8% (*P* < 1.0 × 10^−8^). Upper whisker (Q3 + 1.5 × IQR), Q3, Q2, average, Q1, lower whisker (Q1 – 1.5 × IQR), and all non-outlier data points between lower and upper whiskers were demonstrated on the box plot. The ACD and FMID filters were used to filter in variants of a likely fetal origin. Ref reference, Alt alternative, TP true positive, FP false positive, Q1 lower quartile, Q2 median quartile, Q3 upper quartile, IQR inter-quartile range = Q3 – Q1; ROI regions of interest, ACD allele count distribution, FMID fetal-maternal insert-size distribution, PPV positive predictive value, FF fetal fraction, AF allelic fraction Min *P*-value, minimum of the four *P*-values to examine whether alternative allele fragments are significantly different from the reference allele fragments in length. CDF, the absolute value of the log cumulative distribution function value; NoFLT no variant filter applied.

Unlike fetal chromosomal aberrations involving multiple loci in targeted regions, it requires higher analytical resolution to detect fetal monogenic variants associated with discrete loci. To this end, a new multidimensional algorithm was developed to identify de novo or paternally inherited fetal single gene variants (Fig. 5a, b). The detection of fetal single nucleotide variants (SNVs) can be considered as a process of sampling a population of mixed fetal and maternal DNA molecules. Then the likelihood for an SNV being of fetal origin can be calculated using a beta-binomial distribution based on the number of total DNA molecules detected, FF, and the alternative (alt) allele counts (Materials and methods). We named the filtering step depending on the number of variant allele reads Allele Count Distribution (ACD) filtering (Fig. 5a; Materials and methods). We also found that the fetal cfDNA fragments were ~10 bp on average shorter than the maternal counterpart, while the fragments harboring false positive variants were similar in length to the maternal fragments (Fig. 5c; Supplementary Fig. S10). Fetal-Maternal Insert-size Distribution (FMID) filtering was then established based on the difference of fetal and maternal cfDNA fragment length to discern fetal variants (Fig. 5b). In FMID filtering, a fetal-maternal nearest neighbor insert-size calibration was performed for each NGS read harboring an alt allele at loci where the mother was homozygous for the reference (ref) allele. A binary search was then used to exclude the ref allele read with the closest fragment length to an alt allele read (Fig. 5b). After multiple iterations for all the alt allele reads, the surviving ref allele reads could be regarded maternal. The insert sizes of the remaining reads were then tested under different hypotheses followed by a median comparison to examine whether the alt allele fragments were indeed statistically different or shorter than the ref allele fragments in length (Fig. 5a; Materials and methods). Using the above multidimensional analysis, we examined 28 plasma cfDNA samples and their respective amniocytes collected from pregnant women for 463 genes associated with monogenic disorders. In this test validation set, both common SNPs and rare sequence variants were analyzed (Supplementary Table S2). When the ACD and FMID filters were both applied, the analytical sensitivity and specificity were 99.5% and 99.9%, respectively, improved from the no-filter or ACD filter only method (Fig. 5d, e; Supplementary Table S2). Noteworthily, the ACD and FMID filters were deployed sequentially to filter in variants to avoid over-filtering when variants surviving any of the filtering steps were deemed positive (Fig. 5a; Supplementary Table S3). In summary, by integrating both the allele count and cfDNA fragment length information, we established a new and highly accurate fetal SNV detection method to detect monogenic disorders caused by de novo and paternally inherited sequence variants.

### Evaluation of clinical validity using retrospective pregnant women’s plasma samples

**Fig. 6:**
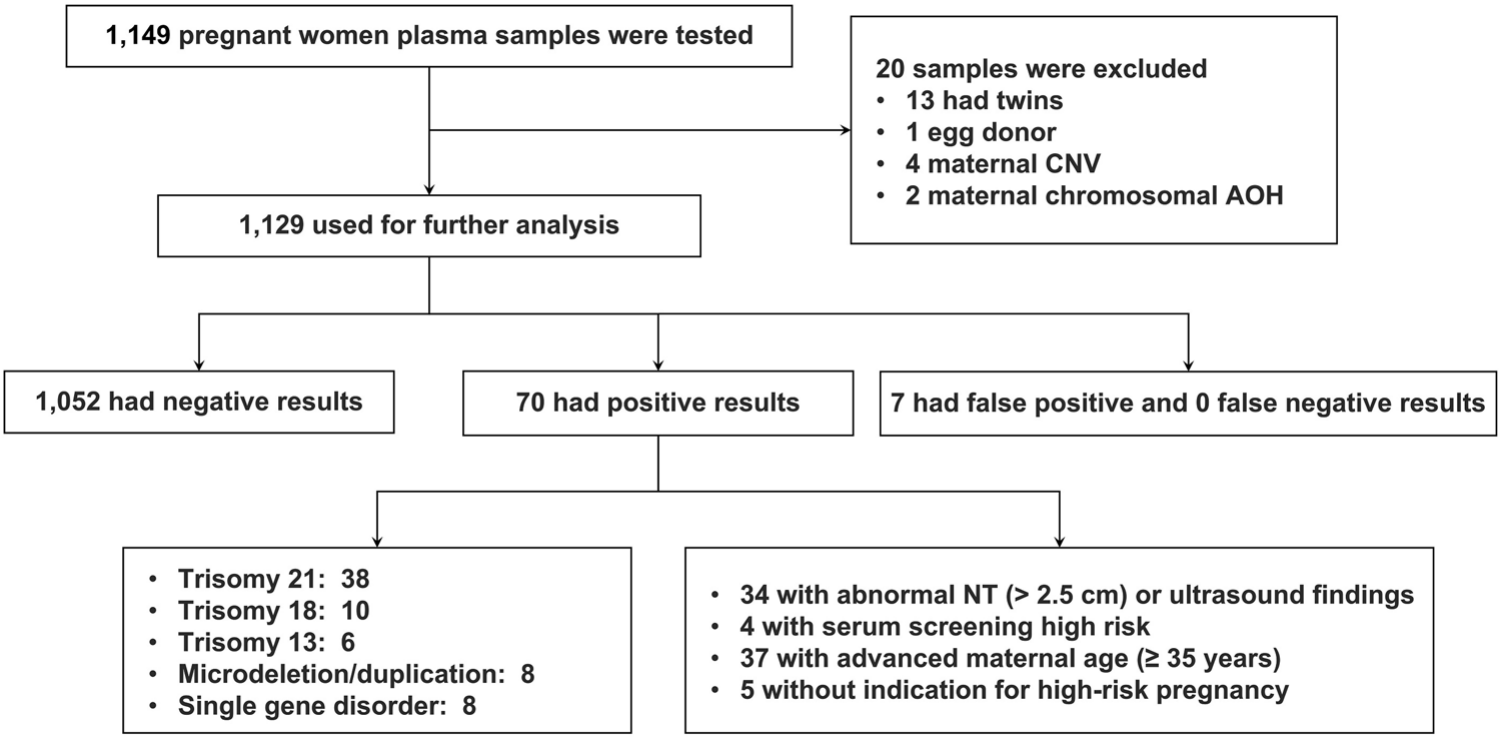
Clinical validation for multiple types of genetic disorders using pregnant women’s plasma samples. A total of 1149 samples collected from pregnant women’s blood were tested. Among them, 20 samples were excluded due to analytical interferences including 13 dizygotic twin pregnancies, one twin pregnancy through egg donor, four with maternal CNV (≥3 Mb), and two with maternal AOH which also failed RD analysis quality control. Next, 1129 samples were subjected to further analysis, and 70 positive cases were identified through the new NIPS method, including 54 aneuploidies, eight MMS and eight cases with monogenic disorders. Clinical information and prenatal findings were collected and shown for 70 positive cases.

As demonstrated above, we developed a new NIPS method that can genetically deconvolute the cfDNA admixture in maternal plasma by querying NGS data associated with fetal and maternal genotypes, RD, SNP linkage, and cfDNA fragment length. Next, we clinically validated this new method using retrospective samples collected from pregnant women. Reproducibility studies including intra-assay, inter-assay, and inter-person were performed, all of which generated consistent results (Supplementary Table S4). Clinical data including testing indication and pregnancy outcome of 1149 cases were collected. Among them, 20 samples were excluded due to detected dizygotic twin pregnancy, egg donor, and maternal CNV/AOH (Fig. 6). The remaining cases were tested for three common chromosomal aneuploidies, eight MMS, and ten monogenic disorders (Materials and methods). For those 1129 samples analyzed, the average gestational age at the time of collection was 15.3 weeks, and the average maternal age was 32.5 years (Supplementary Table S5). There were 426/1129 (37.7%) women with advanced maternal age (≥ 35 years). In the positive cases, 34/70 (48.6%) had increased nuchal translucency or abnormal structural findings (heart structural abnormalities, skeletal abnormalities, etc.); 27/70 (38.6%) had advanced maternal age; 4/70 (5.7%) had abnormal serum screening results; and 5/70 (7.1%) had no known indications (Table 1). For common aneuploidies (T21, T18, and T13), the most frequent indication was advanced maternal age, consistent with previous reports^31^. Two 22q11.2del cases (P55 and P56) were found with abnormal heart development such as tetralogy of Fallot, right aortic arch, persistent left superior vena cava and unclear display of arterial duct, which were consistent with the phenotype of DiGeorge syndrome. MMS cases detected in this study had various or no abnormal prenatal findings (P57 and P61), suggesting the necessity of an expanded NIPS for MMS without specific prenatal indications (Table 1). Eight cases with monogenic variants were detected, all of which were consistent with the prenatal findings seen in these disorders. In subjects P63 and P64 with de novo pathogenic variants in the *PTPN11* and *SOS1* genes, common findings in Noonan spectrum disorder such as thickened pulmonary valve and other anomalies were found in the prenatal ultrasound screening (Table 1). In subjects P65–P70, all fetuses had severe skeletal phenotypes by ultrasound, which were consistent with the findings of pathogenic variants in skeleton-related genes such as *FGFR3*, *FGFR2*, *COL1A1*, *COL1A2*, and *COL2A1* (Table 1).

**Table 1:** Summary of positive clinical cases.

Subject	Gestation age (weeks)	Maternal age (years)	Prenatal finding	Fetal fraction (%)	Screening results	Confirmation study and pregnancy outcome
P1	27.6	42	Abnormal heart development and pleural effusion	6.2	T21	Confirmed by invasive testing
P2	18.7	26	Serum screening high risk	6.5	T21	Confirmed by invasive testing
P3	19.1	35	NT: 3.9 mm	6.9	T21	Confirmed by invasive testing, elective abortion
P4	18.6	37	No ultrasound abnormality	13.0	T21	Confirmed by invasive testing, elective abortion
P5	15.6	33	NT: 4.8 mm, absent nasal bone	10.3	T21	Confirmed by invasive testing
P6	19.3	28	NT: 2.6 mm	13.3	T21	Confirmed by invasive testing, elective abortion
P7	14.9	35	No ultrasound abnormality	8.9	T21	Confirmed by invasive testing
P8	19.3	38	No ultrasound abnormality	8.5	T21	Confirmed by invasive testing
P9	22.4	26	No ultrasound abnormality	9.5	T21	Confirmed by invasive testing, elective abortion
P10	18.1	39	No ultrasound abnormality	11.9	T21	Confirmed by invasive testing, elective abortion
P11	21.1	31	Serum screening high risk	19.3	T21	Confirmed by invasive testing
P12	21.4	38	No ultrasound abnormality	15.3	T21	Confirmed by invasive testing, elective abortion
P13	18.4	38	No ultrasound abnormality	19.2	T21	Confirmed by invasive testing, elective abortion
P14	17.6	39	No ultrasound abnormality	10.4	T21	Confirmed by invasive testing, elective abortion
P15	14.6	28	NT: 4.0 mm	8.3	T21	Confirmed by invasive testing, elective abortion
P16	15.0	27	NT: 3.7 mm	9.1	T21	Confirmed by invasive testing, elective abortion
P17	27.7	27	Abnormal fetal heart development, right heart dominance, mild tricuspid regurgitation, and pericardial effusion	18.8	T21	Confirmed by invasive testing
P18	20.6	28	NT: 2.8 mm	12.3	T21	Confirmed by invasive testing
P19	19.4	27	Serum screening high risk	14.7	T21	Confirmed by invasive testing
P20	16.9	41	No ultrasound abnormality	16.4	T21	Confirmed by invasive testing, elective abortion
P21	15.3	38	No ultrasound abnormality	11.4	T21	Confirmed by invasive testing
P22	13.0	40	NT: 5.8 mm	9.6	T21	Confirmed by invasive testing, elective abortion
P23	19.1	45	Advanced maternal age	15.6	T21	Confirmed by invasive testing, elective abortion
P24	23.3	31	Suspected atrioventricular septal defect with moderate atrioventricular valve regurgitation	18.0	T21	Confirmed by invasive testing
P25	19.3	23	Serum screening high risk	13.4	T21	Confirmed by invasive testing
P26	19.9	26	No ultrasound abnormality	7.5	T21	Confirmed by invasive testing
P27	18.0	36	No ultrasound abnormality	13.7	T21	Confirmed by invasive testing
P28	13.0	37	No ultrasound abnormality	17.0	T21	Confirmed by invasive testing
P29	18.0	37	No ultrasound abnormality	7.6	T21	Confirmed by invasive testing
P30	16.3	38	Advanced maternal age	12.7	T21	Confirmed by invasive testing
P31	21.0	40	No ultrasound abnormality	6.2	T21	Confirmed by invasive testing
P32	14.4	38	No ultrasound abnormality	12.5	T21	Confirmed by invasive testing
P33	24.1	33	No ultrasound abnormality	14.7	T21	Confirmed by invasive testing
P34	13.7	39	No ultrasound abnormality	8.7	T21	Confirmed by invasive testing
P35	22.0	31	No ultrasound abnormality	10.2	T21	Confirmed by invasive testing
P36	13.1	37	No ultrasound abnormality	7.7	T21	Confirmed by invasive testing
P37	12.9	33	NT: 5.2 mm	7.6	T21	Confirmed by invasive testing
P38	23.0	31	No ultrasound abnormality	4.1	T21	Confirmed by invasive testing
P39	17.4	41	No ultrasound abnormality	5.2	T18	Confirmed by invasive testing, elective abortion
P40	14.1	38	NT: 4.9 mm	8.1	T18	Confirmed by invasive testing, elective abortion
P41	18.4	35	No ultrasound abnormality	12.3	T18	Confirmed by invasive testing, elective abortion
P42	18.4	36	No ultrasound abnormality	8.3	T18	Confirmed by invasive testing
P43	20.0	38	No ultrasound abnormality	4.6	T18	Confirmed by invasive testing
P44	13.3	33	NT: 6.5 mm, fetal chest subcutaneous edema	5.3	T18	Confirmed by invasive testing
P45	15.1	33	NT: 3.24 mm	5.5	T18	Confirmed by invasive testing
P46	16.3	37	No ultrasound abnormality	5.9	T18	Confirmed by invasive testing
P47	18.0	25	Growth restriction and umbilical hernia	6.2	T18	Confirmed by invasive testing
P48	15.9	36	No ultrasound abnormality	8.0	T18	Confirmed by invasive testing
P49	19.1	40	Advanced paternal age	8.9	T13	Confirmed by invasive testing
P50	13.9	41	NT: 3.4 mm	5.4	T13	Confirmed by invasive testing, elective abortion
P51	18.1	29	NT: 3.8 mm	9.7	T13	Confirmed by invasive testing
P52	22.0	31	Unclear fetal transparent, upper lip malformation, and heart asymmetry	10.0	T13	Confirmed by invasive testing
P53	18.0	27	NT: 3.0 mm	7.9	T13	Confirmed by invasive testing
P54	13.3	37	NT: 4.4 mm, the inner diameter of the aorta significantly smaller than the pulmonary artery	5.7	T13	Confirmed by invasive testing
P55	23.9	22	Tetralogy of Fallot, persistent left superior vena cava, and unclear display of arterial duct	8.6	22q11.2 deletion	Confirmed by invasive testing, elective abortion
P56	18.4	37	Suspected ventricular septal defect	11.6	22q11.2 deletion	Confirmed by invasive testing, liveborn
P57	18.9	38	No ultrasound abnormality	8.3	22q11.2 deletion	Confirmed by invasive testing, elective abortion
P58	25.0	28	Large foramen ovale	8.5	22q11.2 deletion	Confirmed by invasive testing
P59	23.6	29	Absence of echo in right hemisphere of cerebellum	11.3	15q11.2q12.3 duplication	Confirmed by invasive testing
P60	23.7	36	Heart axis deviating to the left	19.4	4p16.3p14 duplication	Confirmed by invasive testing, elective abortion
P61	19.1	41	No ultrasound abnormality	4.3	15q11.2q13.1 deletion	Confirmed by invasive testing, elective abortion
P62	27.0	32	Lower fetal abdominal bowel echo enhancement	20.0	15q11.2q13.1 deletion	Confirmed by invasive testing
P63	26.3	38	Thickened pulmonary valve with increased pulmonary flow velocity, mild pulmonary valve stenosis, and mild tricuspid regurgitation	15.8	*PTPN11*: c.1502 G > A, variant fraction: 8.6%, Noonan spectrum disorder	Confirmed by invasive testing
P64	31.0	31	Increased head circumference equivalent, lower limb malformation, and enlarged left renal pelvis	30.9	*SOS1*: c.1294T > C, variant fraction: 15.8%, Noonan spectrum disorder	Confirmed by invasive testing
P65	28.1	38	Short femur length	17.0	*FGFR3*: c.1138G > A, variant fraction: 7.0%, achondroplasia	Confirmed by invasive testing
P66	16.4	34	Low ossification of the skull, narrow thoracic cavity, short, long bones, and abnormal limbs	14.3	*COL1A1*: c.4332delC, variant fraction: 6.5%, osteogenesis imperfecta	Confirmed by invasive testing
P67	26.0	30	NT: 0.9 mm, femur length ~3 weeks behind gestation age	14.7	*FGFR3*: c.1138G > A, variant fraction: 8.0%, achondroplasia	Confirmed by invasive testing
P68	13.0	32	Micrognathia and abnormal heart	15.1	*COL2A1*: c.1597 C > T, variant fraction: 8%, type II collagen disorder	Confirmed by invasive testing
P69	24.6	31	Abnormal skull and bilateral fingers and toes	15.6	*FGFR2*: c.755 C > G, variant fraction: 8.3%, Apert syndrome	Confirmed by invasive testing
P70	28.6	27	Short and curved femur	14.1	*COL1A2*: c.3106 G > C, variant fraction: 9.1%, osteogenesis imperfecta	Confirmed by invasive testing

*NT* Nuchal translucency, *T21* trisomy 21, *T18* trisomy 18, *T13* trisomy 13.

Prenatal/postnatal diagnostic results for all cases were retrieved from clinical records to compare with the cfDNA screening results. In 1129 samples analyzed for the validation study, 77 were positive in which 70 were true positive, including 38 T21, ten T18, six T13, eight MMS, and eight monogenic disorder cases (Fig. 6 and Table 1). A total of 1052 cases were tested negative for the disorders screened for. There were zero false negative and seven false positive cases (Fig. 6). The combined sensitivity and specificity for these disorders were 100% (95% CI, 94.9%–100%) and 99.3% (95% CI, 98.6%–99.7%), respectively (Supplementary Table S6). Overall, the validation study demonstrated that this new NIPS approach yielded highly accurate results for the concurrent screening of different types of human genetic disorders.

## Discussion

Accurate and early prenatal diagnosis is essential for the management of the fetal risks for severe genetic diseases. NIPS, through the analysis of fetal cfDNA present in maternal plasma has thus become a prevalent screening approach for common aneuploidies with improved analytical accuracy over conventional biochemical methods or ultrasound screening^32,33^. Significant advances have been made for NIPS by NGS technologies in recent years, which expanded its use for more genetic disorders^12,20,34–40^. However, the inability to reconcile different genetic cues in a single NIPS assay prevents its expansion for the concurrent screening of different types of genetic disorders and improvements on test performance (Supplementary Table S7).

To develop an expanded and enhanced NIPS test in the present work, we applied a top-down design strategy comprised of novel laboratory technologies, comprehensive genomic algorithms, and diseases-specific interpretation analytics. Importantly, a new hybridization-based target enrichment method, termed COATE-seq, was established through allelic probe sequence manipulation (Fig. 1). COATE-seq suppresses the allelic enrichment biases and sequencing variations inherent to conventional methods, which are detrimental to the detection of low-level fetal variants in maternal plasma (Fig. 1). With much improved signal-to-noise ratio, COATE-seq requires only ~20% of the loci used in previous methods to detect common human aneuploidies^11,12^. Next, genomic algorithms were developed to analyze multidimensional cfDNA data including RD, AF, fragment lengths, and linked SNPs to uncover maternal/fetal genotype, meiotic error origins, and meiotic recombination (Figs. 2–5). Overall, the above inventions for important assay reagents, laboratory procedures, and genomic algorithms enabled comprehensive delineation of the fetal genome, which allows the highly accurate detection of fetus-specific variants (Supplementary Table S6).

An important finding resulting from the improved analytical performance of COATE-seq is the discovery of aneuploidy chromosome recombinants in fetal cfDNA (Fig. 4). Common aneuploidies have characteristic parental and meiotic NDJ patterns^26,27,41^. Previous studies showed that the number of meiotic recombination events and the location of crossovers were associated with aneuploidies caused by different meiotic errors^30,42^. Using the NIPS method in this work, we found that 22 T21 cases had a single crossover associated with meiotic NDJ in which most MI NDJ cases had breakpoints within the ~10 Mb of the telomere, while most MII NDJ cases had breakpoints within ~6 Mb of the centromere (Supplementary Fig. S9). This finding is consistent with previous reports showing that recombination breakpoints identified in MI or MII NDJ are in proximity to either telomere or centromere, respectively^30,42^. These results contrast with the location of breakpoints of normal meiotic recombinants which are evenly distributed on both ends of the chromosome^30,42^. With millions of NIPS tests conducted worldwide every year, our method provides a unique tool to investigate the origins of aneuploidies and meiotic recombination from the population perspective.

It should be noted that many frequent and severe recessive monogenic diseases are caused by complex variants residing in genomic regions with homologous sequences (e.g., *SMN1*, *HBA1/A2*, *CYP21A2*, etc.). Recent developments in NGS or long-read sequencing analytics enabled the detection of carriers of the pathogenic variants in these difficult genes^43–45^. However, such variants are not amenable to NIPS tests largely due to the short fragment length of cfDNA. Haplotype-based NIPS for recessive variants has limited utility as pretest information of parental haplotype cannot be readily obtained in population screening. A significant amount of work on current platforms or new technologies are required to develop a practical NIPS test which has superior performance to current carrier testing for those important recessive disorders mentioned above.

Concurrent NIPS for different types of genetic disorders is of obvious clinical value. Monogenic diseases which do not present with gross structural anomalies during early fetal development (e.g., *FGFR3*-related skeletal dysplasia) may be missed in the first-trimester ultrasound screening. By coupling monogenic diseases and chromosomal aberrations for NIPS in early gestation, it allows a more inclusive genetic screen complementing current image-based approaches. While expanding the scope of NIPS has sensible benefits, evidence-based studies for such screening are yet to be performed to prove its clinical utility. In future studies, it will be important to address key issues such as disease inclusion criteria for screening, proper testing indications, and reporting or genetic counseling strategies for variants of phenotypic variability or incomplete penetrance. In addition, pregnancy management options and their outcomes need to be evaluated for individuals affected by either positive or negative screening results to weigh the benefits and risks of offering a comprehensive NIPS test.

Overall, we presented here a comprehensive NIPS approach with improvements in assay performance, underlying technical innovations, and new cfDNA analytical algorithms. This new approach overcomes the limitations of current methods, which do not concurrently screen chromosomal and monogenic disorders and are confounded by multiple gestations, and maternal CNV and AOH. This method shows its potential in clinical application, especially enabling a more accurate prenatal screening on a broad spectrum of genetic diseases and providing valuable assistance for risk evaluation and timely management of pregnancy.

## Materials and methods

### Study subjects and ethics protocol

A total of 1182 samples from human subjects were included in the clinical validation study. This study was approved by the institutional review boards of China International Peace Maternity and Child Health Hospital (GKLW2019-52) and Obstetrics and Gynecology Hospital of Fudan University (2020-178). Informed consent was obtained from all study participants. This study collected leftover samples from subjects who underwent amniocentesis or chorionic villus sampling for standard prenatal diagnosis. Maternal plasma was collected before their invasive prenatal diagnosis procedures. The results from karyotyping, microarray-based comparative genomic hybridization, and/or sequencing data were collected for all cases with available pregnancy outcome data for the validation study.

### cfDNA extraction and NGS

The maternal plasma was separated through a two-step centrifugation process. At least 0.8 mL maternal plasma was first separated from the whole blood by centrifugation of the collection tube at 1600× *g* for 15 min at 4 °C. Then the plasma was centrifuged at 16,000× *g* for 10 min at 4 °C. cfDNA extraction was performed using Magnetic Serum/Plasma Circulating DNA Maxi Kit (Tiangen, China). For the NGS library construction, the cfDNA was end-repaired using the manufacturer’s protocol (Nanodigbio, China) followed by ligation at 20 °C for 15 min using adapters with unique molecular indexes. The sample barcode was introduced by PCR: 98 °C for 2 min, then nine cycles at 98 °C for 15 s, 60 °C for 30 s, and 72 °C for 30 s followed by a final extension at 72 °C for 2 min. The PCR products were then quantified using Qubit^TM^ 1× dsDNA HS Assay Kits (Invitrogen, United States). All the cfDNA extracted from each sample was used for library construction, which must be at least 400 ng after PCR amplification before proceeding to the next step for target enrichment. 12–36 samples were then pooled together for target enrichment at 65 °C or 68 °C for 16 h. Hybridization probes were added to the pooled DNA using the manufacturer’s protocol (Heristar LLC, United States). The recovered DNA was washed and purified using Dynamag-270 magnetic beads (Invitrogen, United States). Another PCR was performed to generate sequencing library: 98 °C for 2 min, then 12 cycles at 98 °C for 15 s, 60 °C for 30 s, and 72 °C for 30 s and a final extension at 72 °C for 2 min. Single-stranded circular DNA libraries were prepared by MGI-Easy Circularization Kit (MGI, China). Circular DNA was produced to generate DNA nanoballs by rolling circle amplification using the manufacturer’s protocol (MGI, China). The concentration of sequencing library was quantified by Qubit using Qubit ssDNA Assay Kits (Invitrogen, United States). The final DNA library was sequenced on MGISEQ-2000 (MGI, China) using 2× 100 paired-end mode.

### COATE probe design

Probes were designed to target SNPs on the entire or critical regions of chromosomes 1, 2, 4, 5, 8, 13, 15, 18, 21, 22, X, and Y to screen for T13, T18, T21, and sex chromosome aneuploidies or 1p36 microdeletion (chr1:800,095-12,734,180), 2q33.1 microdeletion (chr2:196,535,270- 202,435,277), Wolf-Hirschhorn syndrome (chr4:425,435-2,108,509), Cri du Chat syndrome (chr5:10,001-18,399,891), Langer-Giedion syndrome (chr8:116,700,000-126,300,000), Jacobsen syndrome (chr11:114,629,279-135,076,622), Prader-Willi syndrome/Angelman syndrome (chr15:23,334,675-28,323,850), and DiGeorge syndrome (chr22:17,322,843-21,118,912). All chromosome coordinates are GRCh38. The probes must reside in targeted regions where the GC content ranges from 30% to 70%. Additional probes were designed to target the coding sequences of genes including *COL1A1*, *COL2A1*, *FGFR2*, *FGFR3*, *COL1A2*, *PTPN11*, *RAF1*, *RIT1*, and *SOS1* which are associated with frequent human dominant monogenic disorders. To reduce the probe hybridization bias between the reference and alternative alleles, one of four possible nucleotides (A, C, G, and T) is selected at the locus corresponding to the target SNP (Fig. 1b). Such probes are expected to have the minimum melting temperatures (*T*m) difference for their pairing with reference allele *N*_ref_ and alternative allele *N*_Alt_. The Nearest Neighbor model is used to calculate the Tm^46^.

$$\mathop {{\rm{argmin}}}\limits_N \left\{ {\left| {T_m\left( {N_{\rm{ref}}} \right) - T_m\left( {N_{\rm{alt}}} \right)} \right|} \right\},N \in \left\{ {A,C,G,T} \right\}$$

### The FF calculation

At any biallelic locus, there are two possible euploid maternal-fetal genotype combinations including AA-AB and BB-AB (A: alternative allele, B: reference allele) informative for the FF calculation. At SNP *i*, let *N* be the total NGS reads of the A and B alleles, and *NA*_*i*_ be the number of A allele reads. At the loci where the mother is homozygous (AA or BB) and the fetus is heterozygous (AB), the locus-specific FF (denoted by *FF*_*AAi*_ or *FF*_*BBi*_) can be computed.

$$FF_{AA_i} = 2\left( {1 - \frac{{NA_i}}{N}} \right)$$

$$FF_{BB_i} = \frac{{2NA_i}}{N}$$

$$N = NA_i + NB_i$$

Denote *FF*_*AA*_ and *FF*_*BB*_ the median of the FFs calculated for all informative SNPs, the sample *FF* is then calculated.

$$FF = \left( {FF_{AA} + FF_{BB}} \right)/2$$

A RD-based method was used to quantify Y chromosome dosage for FF in pregnancies with male fetus. Specifically, uniquely mapped reads to the Y and X chromosomes were counted followed by a normalization step. Then the ratio of median counts of normalized reads for the Y and X chromosomes was used to estimate the FF.

$$FF = RDchrY/\left( {RDchrY + RDchrX} \right)$$

### The locus-specific loglikelihood of different aneuploid states

The AF of SNP *i* can be considered as the probability of sampling the A allele (*pA*_*i*_). Let *Ni* be the total NGS reads of the A and B alleles at the SNP *i* and *NA*_*i*_ be the number of A allele reads. The beta-binomial distribution is used to calculate the likelihood of sampling A allele with parameters based on different ploidy hypotheses. For the beta function, α is set at an empirical value of 3000. The AFs (or *pA*_*i*_) can be computed when fetal genotypes in different ploidy state and FF are accounted for (Supplementary Table S8). Then, the *β* is calculated.

$$\beta = \alpha /pA_i-\alpha$$

The *pA*_*i*_ under different H, was derived from a linear combination of the conditional beta-binomial distributions which were weighted by the multinomial factor π_k_ and $\mathop {\sum }\limits_k \pi k = 1$ using a modified method from a previous study^47^. Finally, with the depth and A allele reads, the likelihood can be computed by the beta-binomial distribution as follows:

$$p\left( {NAi|N,\alpha ,\beta ,FF,H} \right) = \mathop {\sum }\limits_k \pi _k\left( {\begin{array}{*{20}{c}} N \\ {NAi} \end{array}} \right)\frac{{B\left( {NAi + \alpha ,N - NAi + \beta } \right)}}{{B\left( {\alpha ,\beta } \right)}}$$

$$H\,\upepsilon\, \left\{ {D,MI,MII,PI,PII,LM,LP} \right\}$$

where *D*, *MI*, *MII*, *PI*, *PII*, *LM* and *LP* are disomy, trisomy (maternal meiosis I nondisjunction), trisomy (maternal meiosis II nondisjunction), trisomy (paternal meiosis I nondisjunction), trisomy (paternal meiosis II nondisjunction), monosomy (maternal meiosis nondisjunction) and monosomy (paternal meiosis nondisjunction), respectively. The following formulas are used to calculate *π*_*k*_:

$$\pi _k = \mathop {\sum}\limits_{PAT_i} {p\left( {FET} \right) \ast p\left( {PAT_i} \right)}$$

$$PAT_{ki} \in \left\{ {AA,AB,BB} \right\}$$

where the *p*(*PAT*) is the probability of the paternal genotype at the SNP *i* locus and the *p* is the population frequency of SNP *i* and the *p*(*FET*) is the probability of a specific fetal genotype in different euploid and aneuploid states when a familial trio is analyzed following the Mendelian inheritance principle (Supplementary Table S9). The *p(PAT)* is computed using the Hardy-Weinberg equilibrium.

$$p\left( {AA} \right) = p^2$$

$$p\left( {AB} \right) = 2p\left( {1 - p} \right)$$

$$p(BB) = (1 - p)^2$$

After *pA*_*i*_ of all SNPs on each target chromosome were calculated, a Hampel filter was used to detect and remove outliers.

### Maximum likelihood of fetal chromosome ploidy

When there is no meiotic recombination, the fetus should always inherit all the SNPs from an entire parental chromosome. Let *m* be the total number of informative SNPs on the target chromosome. Then the maximum likelihood of fetal chromosome ploidy can be computed by taking the aggregate likelihoods of ploidy state at each informative locus. A particular fetal aneuploidy state could be established in which the respective *∆L* value is negative and the lowest among all possible *H*:

$$\Delta L = \mathop {\sum}\limits_{i = 1}^M {\left( {\log \left( {Lp\left( {D_i} \right)} \right) - \log \left( {p\left( {H_i} \right)} \right)} \right)}$$

$$H\,\upepsilon\, \left\{ {MI,MII,PI,PII,LM,LP} \right\}$$

where the *∆L* is sum of the locus-specific loglikelihood difference between a euploidy and an aneuploidy state, denoted by *p(D*_*i*_*)* and *p(H*_*F*_*)*, respectively.

If there is meiotic recombination associated with aneuploidy, the fetus inherits the SNPs on a recombinant chromosome derived from the paired homologous chromosomes in the chiasma stage. The maximum likelihood of chromosome aneuploidy with one recombination is computed:

$$\Delta L = \min \left( {\mathop {\sum}\limits_1^k {\left( {{\text{log}}\left( {LD_i} \right) - \log \left( {LH1_i} \right)} \right)} + \mathop {\sum}\limits_{k + 1}^M {\left( {\log \left( {LD_i} \right) - \log \left( {LH2_i} \right)} \right)} } \right)$$

$$\{ H1,H2|H1\mathop { \cup }\nolimits H2 \in \left\{ {{{{\mathrm{MI,MII}}}}} \right\}\,{{{\mathrm{or}}}}\,\,H1\mathop { \cup }\nolimits H2 \in \{ {{{\mathrm{PI,PII}}}}\} \}$$

Similarly, the maximum likelihood is computed with two breakpoints a chromosome:

$$\Delta L\left( {H1,H2} \right) = \min \left( \begin{array}{l}\mathop {\sum}\limits_1^{b1} {\left( {\log \left( {LD_i} \right) - \log \left( {LH1_i} \right)} \right)} \\ + \mathop {\sum}\limits_{b1 + 1}^{b2} {\left( {\log \left( {LD_i} \right) - \log \left( {LH2_i} \right)} \right)} \\ + \mathop {\sum}\limits_{b2 + 1}^M {\left( {\log \left( {LD_i} \right) - \log \left( {LH1_i} \right)} \right)} \end{array} \right)$$

$$\left\{ {H1,H2|H1\mathop { \cup }\nolimits H2 \in \left\{ {{{{\mathrm{MI,MII}}}}\;{{{\mathrm{or}}}}\;H1\mathop { \cup }\nolimits H2 \in \left\{ {{{{\mathrm{PI,PII}}}}} \right\}} \right.} \right\}$$

### Chromosome CNV analysis using the RD data

The NGS raw reads were aligned to hg38 followed by unique molecular index (UMI)-based deduplication to suppress PCR amplification artifacts. Unmapped or ambiguously mapped reads were excluded from the coverage-based copy number analysis. For data normalization, the RD medians for each target chromosome (e.g., chr21, chr18, chr13, chr22, etc.) were found. Then, the median of the above chromosome-specific RD medians was used as the normalized sample coverage. SNP-specific RD was scaled proportionally to the normalized sample average for inter-sample comparison. A Hampel filter was used to remove loci with skewed RD which had > 3 standard deviations of the moving average. Principle component analysis (PCA) was used to remove the primary components of the reads which were independent of copy number changes^48^. This PCA-adjusted RD was then used for *Z*-score calculation_._ The entire dataset was divided into two groups. One group of 405 samples was used for method development and the other group of 724 samples was used for validation. The ratio of each target chromosome to the total reads of reference chromosomes within each sample was calculated for comparison. *Z*-score was calculated based on all samples in the same processing batch:

$$Z_i = \frac{{R_i - \mu _i}}{{\sigma _i}}$$

where *R*_*i*_, *µ*_*i*_, and *σ*_*i*_ are the ratio, average and variance of the ratio of target region, respectively. After multiple in silico experiments of pair-wise parameters for FF, RD, and the number of informative loci, we determined that the FF-RD product must reach 48 and the total number of informative SNPs must be > 60 to guarantee a test sensitivity ≥ 95%.

### The detection of maternal CNV and dizygotic twins

Maternal CNV is detected based on both the RD and the SNP AF data. The RD of each SNP is normalized by dividing it with the median depth of reference chromosome SNPs. Next, the median and standard deviation of normalized RD of each SNP is calculated, followed by *Z*-score calculation which is subjected to a smoothing step using a mean-shift algorithm. For each cluster, if the center has a *Z*-score > 3 or < –3, then a potential maternal CNV is called. This cut-off is set based on the assumption that the FF is usually < 0.5, a level at which the expected *Z*-score for each SNP is between –2.5 and 2.5, even when the fetus has a chromosome copy number gain or loss. For samples with maternal mosaic CNV, if the mosaic level is < 0.6, it is beyond the detection limit using the *Z*-score method. To reduce the false positive calls, the SNP AFs in regions with potential maternal CNV are also examined. For samples that have a maternal deletion, the heterozygous SNP AF is > 0.6 or < 0.4. For samples that have a maternal duplication, the heterozygous AF is between 0.4 and 0.6. The maternal CNV call is rejected if there is any conflicting result from the SNP AF data. Dizygotic twin pregnancy is determined by the increased number of fetal SNPs detected and the variation of the AF of fetal SNPs (Fig. 3g).

### The detection of meiotic homologous recombination for aneuploidies

The value of SNP AF is dictated by the specific combination of the maternal and fetal genotypes associated with different ploidy states (Supplementary Fig. S6a). In trisomies caused by maternal meiotic errors, multiple consecutive fetal homozygous SNPs (AAA or BBB) at the loci where the mother is heterozygous are indicative of MII NDJ (Supplementary Fig. S6a). Similarly, when the homologs are transmitted in MI NDJ, no extensive stretch of fetal homozygous SNPs should be present at the loci where the mother is heterozygous (Supplementary Fig. S6a). Therefore, the presence of both MI NDJ and MII NDJ fetal genotypes are suggestive of a recombinant event (Supplementary Fig. S6b, c). In MI NDJ causing fetal trisomy, at the maternal heterozygous loci (AB), the probability of fetus being heterozygous (ABA or ABB) is 1 (Supplementary Fig. S5). Therefore, when fetal homozygosity (genotype AAA or BBB) disrupting the MI NDJ pattern is seen, it can only be explained by an event of meiotic recombination. In MII NDJ, the probability of a fetus being heterozygous (genotype AAB or ABB) at a maternal heterozygous locus is 0.5 (Supplementary Fig. S5). Assuming all loci are transmitted independently and there is no recombination, the probability of 10 consecutive informative SNPs showing only fetal heterozygosity is 2^−10^ in MII. Therefore, we use a cut-off of 10 continuous fetal heterozygous loci to determine whether there is a MI NDJ.

### Monogenic variant detection

To screen for fetal SNVs in the regions of interest, repeat region filter was applied for all repeat region marked by RepeatMasker and excluded from the benchmark validation set. All fetal SNVs of cfDNA samples and related germline SNPs of amniocyte samples in the qualified region were identified by a modified BWA-GATK based in-house pipeline. For all amniocyte samples, germline SNPs with depth ≥ 100 and AF ≥ 30% were utilized as golden standard for cfDNA fetal SNV calling results. As for cfDNA samples, sites with depth ≥ 200 or AF ≥ 1% were used for the benchmark process.

ACD filter was established to identify paternally inherited or de novo fetal SNVs based on the expected alternative allele counts. The beta-binomial distribution was used to calculate the likelihood of a variant being from a non-fetal origin. When the absolute value of the log cumulative distribution function (CDF) value is between –10 and –0.001, the variant is considered positive. Note that the variants not falling in this range can still be considered positive if they satisfy downstream fragment size-based inclusion filters. Beta-binomial cumulative distribution function:

$$F\left( {x\left| {n,\alpha ,\beta } \right.} \right) = P\left( {X \le x} \right) = \mathop {\sum}\limits_{i = 0}^{\lfloor{x}\rfloor} {\left( {\begin{array}{*{20}{c}} n \\ i \end{array}} \right)\frac{{B\left( {i + \alpha ,n - i + \beta } \right)}}{{B\left( {\alpha ,\beta } \right)}}}$$

$$\alpha = \frac{{d_vmf}}{{2d_{avg}}}$$

$$\beta = \frac{{d_vm\left( {2 - f} \right)}}{{2d_{avg}}}$$

where the *x* is the alternative allele depth for a certain variant; *n* is the total sequencing depth for a certain variant; *α* is the effective alternative allele DNA molecule count for a certain variant and sample before PCR; *β* is the effective reference allele DNA molecule count for a certain variant and sample before PCR; *d*_*avg*_ is the sample average effective sequencing depth; *d*_*v*_ is the variant effective sequencing depth; *m* is the empirical value of total effective DNA molecule count before PCR; and *f* is the FF.

FMID filter was established to identify paternally inherited or de novo fetal SNVs based on the fragment lengths. Insert-size was first smoothed for all abnormally short and long fragments, which were set between 20 and 600 bp to avoid alignment artifacts. Next, insert-sizes of reference and alternative allele supporting reads were sorted in an ascending order. For each insert-size from the alternative allele supporting group, a modified binary search algorithm was used to locate the closet insert-size value in the reference allele supporting group. The fragment with the nearest neighbor insert-size value from the reference allele supporting group was then excluded. Specifically, the same number of fragments carrying reference allele that had similar size of those carrying alternative allele were excluded to better distinguish maternal- and fetal-origin fragments (Fig. 5b). After multiple iterations for all the alternative allele reads, the surviving reference allele reads can be regarded as being from the maternal origin. Then insert-sizes of the remaining reads were tested under four different hypotheses followed by a median comparison to test whether the alternative allele fragments were indeed statistically different and shorter than the reference allele fragments. To compare the difference of fragment length between the alternative allele reads and reference allele reads, the *P*-values of four hypotheses including Welch’s *t*-test, Kolmogorov–Smirnov test, Kruskal–Wallis *H*-test with correction for ties and Mann–Whitney *U*-test with correction for ties were calculated. A variant was considered potentially positive when the minimum of the four *P*-values (MinP) was ≤ 0.001. After testing whether the fragment lengths were significantly different, another comparison was performed to test whether the fragment lengths were shorter in those alternative allele reads than those reference allele reads. When the median of the alternative allele reads was shorter than the median of the reference allele reads, the variant was considered potentially positive (*K* > 0.5; Fig. 5a and Supplementary Fig. S10). Note that a variant was filtered out only when all the above exclusion filtering criteria were met (Fig. 5a).

### Lower detection of limit

In 104 samples using mixed match maternal and fetal DNA including T21, T18, T13, 22q11.2del, and *FGFR3*: c.1138G > A with fetal percentage ranging from 4.0% to 5.8%, the overall detection rate for the target disease at case level was 99.1%. Therefore, we concluded that the lower detection limit of this test was at 4.0%.

### The analysis workflow

MGISEQ-2000 sequencers with the PE100 chemistry were used to sequence batched cfDNA samples followed by the secondary analysis on a dual AMD EPYC7713 (128 core) with 768 G memory server. The secondary analysis lasted ~5–6 h per run, which included FASTQ quality control, deduplication through UMI processing, alignment, making consensus reads, BAM processing, variant calling, variant filtering, and variant annotation. The variant interpretation was performed following guidelines from the American College of Medical Genetics. Maternal genotype was obtained through maternal cfDNA testing while paternal testing was not required for reporting pathogenic or likely pathogenic variants in this study.

## Supplementary information

Supplementary information

## Data Availability

These authors declare that all essential data supporting the conclusion of the study as well as detailed assay protocols and analytical algorithms are within the paper and Supplementary information. All the disease-causing sequence variants and the key phenotypes found in the subjects can be found at the ClinVar database (http://www.ncbi.nlm.nih.gov/clinvar/variation/). Subjects’ identifiable information (including their genomic sequencing data) is kept in our clinical laboratory, which is a HIPAA-compliant environment, to protect subjects’ privacy. Non-identifiable sequencing data (e.g., individual variant sequencing data generated by locus-specific sequencing) can be provided by the authors upon request. The customized computational codes as well as their training dataset are available upon request. Software is free for academic use and can be licensed for commercial use.

## References

[1] Mai CT (2019). National population-based estimates for major birth defects, 2010-2014. Birth Defects Res..

[2] Brent RL (2004). Environmental causes of human congenital malformations: the pediatrician’s role in dealing with these complex clinical problems caused by a multiplicity of environmental and genetic factors. Pediatrics.

[3] Amberger JS, Bocchini CA, Scott AF, Hamosh A (2019). OMIM.org: leveraging knowledge across phenotype-gene relationships. Nucleic Acids Res..

[4] Antonarakis SE (2019). Carrier screening for recessive disorders. Nat. Rev. Genet..

[5] Chiu RW (2008). Noninvasive prenatal diagnosis of fetal chromosomal aneuploidy by massively parallel genomic sequencing of DNA in maternal plasma. Proc. Natl. Acad. Sci. USA.

[6] Liang D (2019). Clinical utility of noninvasive prenatal screening for expanded chromosome disease syndromes. Genet. Med..

[7] Chitty LS (2015). Non-invasive prenatal diagnosis of achondroplasia and thanatophoric dysplasia: next-generation sequencing allows for a safer, more accurate, and comprehensive approach. Prenat. Diagn..

[8] Zhang J (2019). Non-invasive prenatal sequencing for multiple Mendelian monogenic disorders using circulating cell-free fetal DNA. Nat. Med..

[9] Flori E (2004). Circulating cell-free fetal DNA in maternal serum appears to originate from cyto- and syncytio-trophoblastic cells. Case report. Hum. Reprod..

[10] Ashoor G, Syngelaki A, Poon LC, Rezende JC, Nicolaides KH (2013). Fetal fraction in maternal plasma cell-free DNA at 11-13 weeks’ gestation: relation to maternal and fetal characteristics. Ultrasound Obstet. Gynecol..

[11] Zimmermann B (2012). Noninvasive prenatal aneuploidy testing of chromosomes 13, 18, 21, X, and Y, using targeted sequencing of polymorphic loci. Prenat. Diagn..

[12] Hall MP (2014). Non-invasive prenatal detection of trisomy 13 using a single nucleotide polymorphism- and informatics-based approach. PLoS ONE.

[13] Curnow KJ (2015). Detection of triploid, molar, and vanishing twin pregnancies by a single-nucleotide polymorphism-based noninvasive prenatal test. Am. J. Obstet. Gynecol..

[14] Snyder MW (2015). Copy-number variation and false positive prenatal aneuploidy screening results. N. Engl. J. Med..

[15] Grati FR (2014). Fetoplacental mosaicism: potential implications for false-positive and false-negative noninvasive prenatal screening results. Genet. Med..

[16] Benn P, Rebarber A (2021). Non-invasive prenatal testing in the management of twin pregnancies. Prenat. Diagn..

[17] Zhou X (2017). Contribution of maternal copy number variations to false-positive fetal trisomies detected by noninvasive prenatal testing. Prenat. Diagn..

[18] Pergament E (2014). Single-nucleotide polymorphism-based noninvasive prenatal screening in a high-risk and low-risk cohort. Obstet. Gynecol..

[19] Samango-Sprouse C (2013). SNP-based non-invasive prenatal testing detects sex chromosome aneuploidies with high accuracy. Prenat. Diagn..

[20] Teder H (2019). Computational framework for targeted high-coverage sequencing based NIPT. PLoS ONE.

[21] Chan KC (2004). Size distributions of maternal and fetal DNA in maternal plasma. Clin. Chem..

[22] Snyder MW, Kircher M, Hill AJ, Daza RM, Shendure J (2016). Cell-free DNA comprises an in vivo nucleosome footprint that informs its tissues-of-origin. Cell.

[23] Liang B (2018). Enrichment of the fetal fraction in non-invasive prenatal screening reduces maternal background interference. Sci. Rep..

[24] Artieri CG (2017). Noninvasive prenatal screening at low fetal fraction: comparing whole-genome sequencing and single-nucleotide polymorphism methods. Prenat. Diagn..

[25] Sherman SL (1994). Non-disjunction of chromosome 21 in maternal meiosis I: evidence for a maternal age-dependent mechanism involving reduced recombination. Hum. Mol. Genet..

[26] Bugge M (1998). Non-disjunction of chromosome 18. Hum. Mol. Genet..

[27] Bugge M (2007). Non-disjunction of chromosome 13. Hum. Mol. Genet..

[28] Vaitiekunas P, Crane-Robinson C, Privalov PL (2015). The energetic basis of the DNA double helix: a combined microcalorimetric approach. Nucleic Acids Res..

[29] Mikwar M, MacFarlane AJ, Marchetti F (2020). Mechanisms of oocyte aneuploidy associated with advanced maternal age. Mutat. Res. Rev. Mutat. Res..

[30] Lamb NE (1997). Characterization of susceptible chiasma configurations that increase the risk for maternal nondisjunction of chromosome 21. Hum. Mol. Genet..

[31] Nicolaides KH (2011). Screening for fetal aneuploidies at 11 to 13 weeks. Prenat. Diagn..

[32] Allyse M (2015). Non-invasive prenatal testing: a review of international implementation and challenges. Int J. Women’s Health.

[33] Sentilhes L, Salomon LJ, Vayssiere C (2015). Cell-free DNA analysis for noninvasive examination of trisomy. N. Engl. J. Med..

[34] Kitzman JO (2012). Noninvasive whole-genome sequencing of a human fetus. Sci. Transl. Med..

[35] Yu SC (2014). Size-based molecular diagnostics using plasma DNA for noninvasive prenatal testing. Proc. Natl. Acad. Sci. USA.

[36] Sun K (2018). Size-tagged preferred ends in maternal plasma DNA shed light on the production mechanism and show utility in noninvasive prenatal testing. Proc. Natl. Acad. Sci. USA.

[37] Koumbaris G (2019). Targeted capture enrichment followed by NGS: development and validation of a single comprehensive NIPT for chromosomal aneuploidies, microdeletion syndromes and monogenic diseases. Mol. Cytogenet..

[38] Rabinowitz T (2019). Bayesian-based noninvasive prenatal diagnosis of single-gene disorders. Genome Res..

[39] Serpas L (2019). Dnase1l3 deletion causes aberrations in length and end-motif frequencies in plasma DNA. Proc. Natl. Acad. Sci. USA.

[40] Che H (2020). Noninvasive prenatal diagnosis by genome-wide haplotyping of cell-free plasma DNA. Genet. Med..

[41] Freeman SB (2007). The National Down Syndrome Project: design and implementation. Public Health Rep..

[42] Oliver TR (2014). An examination of the relationship between hotspots and recombination associated with chromosome 21 nondisjunction. PLoS ONE.

[43] Feng Y (2017). The next generation of population-based spinal muscular atrophy carrier screening: comprehensive pan-ethnic SMN1 copy-number and sequence variant analysis by massively parallel sequencing. Genet. Med..

[44] He J (2017). Next-generation sequencing improves thalassemia carrier screening among premarital adults in a high prevalence population: the Dai nationality, China. Genet. Med..

[45] Liu Y (2022). Comprehensive analysis of congenital adrenal hyperplasia using long-read sequencing. Clin. Chem..

[46] SantaLucia, J. Jr. A unified view of polymer, dumbbell, and oligonucleotide DNA nearest-neighbor thermodynamics. *Proc. Natl. Acad. Sci. USA* **95**, 1460–1465 (1998).. 10.1073/pnas.95.4.1460 PMC19045 9465037

[47] Jiang P (2012). FetalQuant: deducing fractional fetal DNA concentration from massively parallel sequencing of DNA in maternal plasma. Bioinformatics.

[48] Fromer M (2012). Discovery and statistical genotyping of copy-number variation from whole-exome sequencing depth. Am. J. Hum. Genet..

