# Supplementary material for: Genetic deconvolution of fetal and maternal cell-free DNA in maternal plasma enables next-generation non-invasive prenatal screening
Source: Cell Discov. 2022 Oct 13;8:109. doi: 10.1038/s41421-022-00457-4 (PMC9562363; doi:10.1038/s41421-022-00457-4)
Supplement: Supplementary file 1 — Supplementary information [file 41421_2022_457_MOESM1_ESM.pdf]

Genetic deconvolution of fetal and maternal cell-free DNA in maternal plasma enables next-generation non-invasive prenatal screening

Chenming Xu[1,2,*,#], Jianli Li[3,#], Songchang Chen[1,2,4,#], Xiaoqiang Cai[3,#], Ruilin Jing[3], Xiaomei Qin[3], Dong Pan[3], Xin Zhao[3], Dongyang Ma[3], Xiufeng Xu[3], Xiaojun Liu[3], Can Wang[3], Bingxin Yang[2], Lanlan Zhang[2], Shuyuan Li[2], Yiyao Chen[2], Nina Pan[2], Ping Tang[5], Jieping Song[6], Nian Liu[6], Chen Zhang[1,2], Zhiwei Zhang[3], Xiang Qiu[7], Weiliang Lu[7], Chunmei Ying[7], Xiaotian Li[7], Congjian Xu[7], Yanlin Wang[2], Yanting Wu[1,8,*], He-Feng Huang[1,2,8,9*], Jinglan Zhang[1,2,3,*]

[1]Obstetrics and Gynecology Hospital, Institute of Reproduction and Development, Fudan University, Shanghai, China, 200011

[2]International Peace Maternity and Child Health Hospital, School of Medicine, Shanghai Jiao Tong University, Shanghai, China, 200030

[3]Beijing BioBiggen Technology Co., Ltd., Beijing, China, 100176

[4]State Key Laboratory of Genetic Engineering and MOE Engineering Research Center of Gene Technology, School of Life Sciences, Fudan University, Shanghai, China, 200433

[5]Jiaxing Maternity and Child Health Care Hospital, Jiaxing, China, 314051

[6]Medical Genetics Center, Maternal and Child Health Hospital of Hubei Province, Wuhan, Hubei, China, 430070

[7]Obstetrics and Gynecology Hospital of Fudan University, Shanghai, China, 200011

[8]Shanghai Key Laboratory of Embryo Original Diseases, Shanghai, China, 200030

[9]Research Units of Embryo Original Diseases, Chinese Academy of Medical Sciences (No.2019RU056), Shanghai, China, 200011

[#]These authors equally contribute to this work.

*Correspondence: Jinglan Zhang (jinglanzhang@foxmail.com), Chenming Xu (chenming_xu2006@163.com), He-Feng Huang (huanghefg@hotmail.com), Yanting Wu (yanting_wu@163.com).

Supplementary Figures

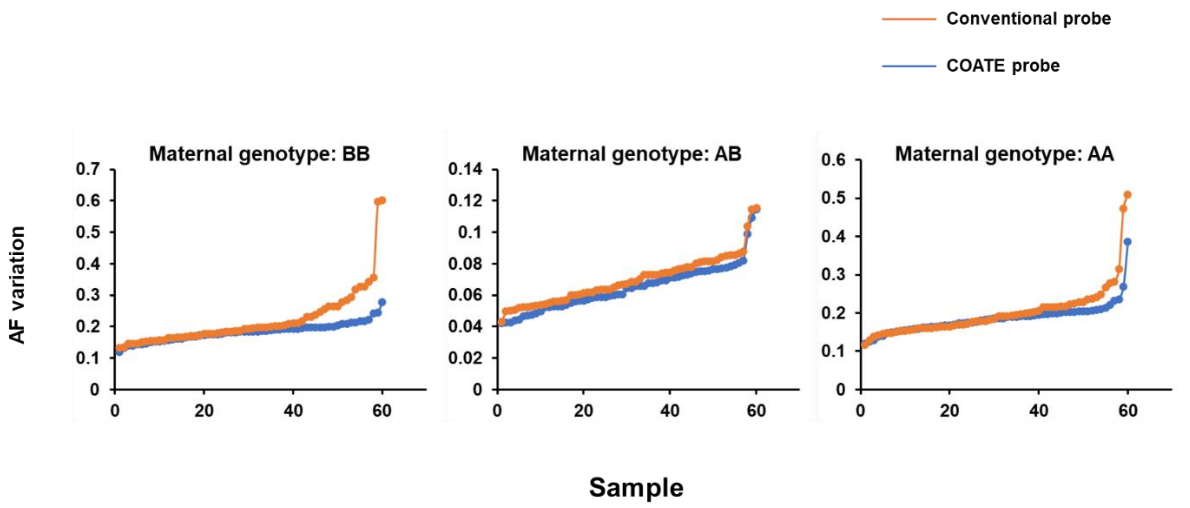

Supplementary Fig. S1 The comparison of the measured variation of SNP allelic fraction using the conventional and COATE probes. The coefficient of variation of the measured SNP allelic fraction for all SNPs where the maternal genotype is BB, AA, and AB.

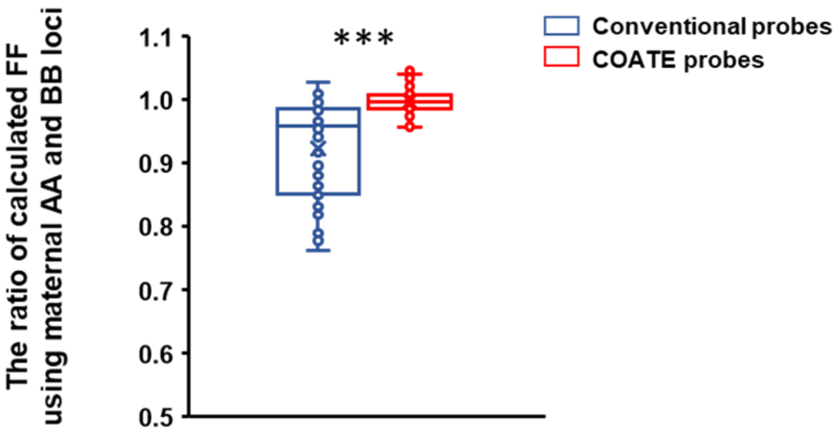

Supplementary Fig. S2 The calculated fetal fraction using conventional and COATE probes. The ratio of fetal fraction calculated by fetal heterozygous SNPs at maternal AA loci to BB loci was 0.923 ± 0.078 (n=60) using conventional probes while it was 0.998 ± 0.020 (n=60) using the COATE probes (p<0.0001).

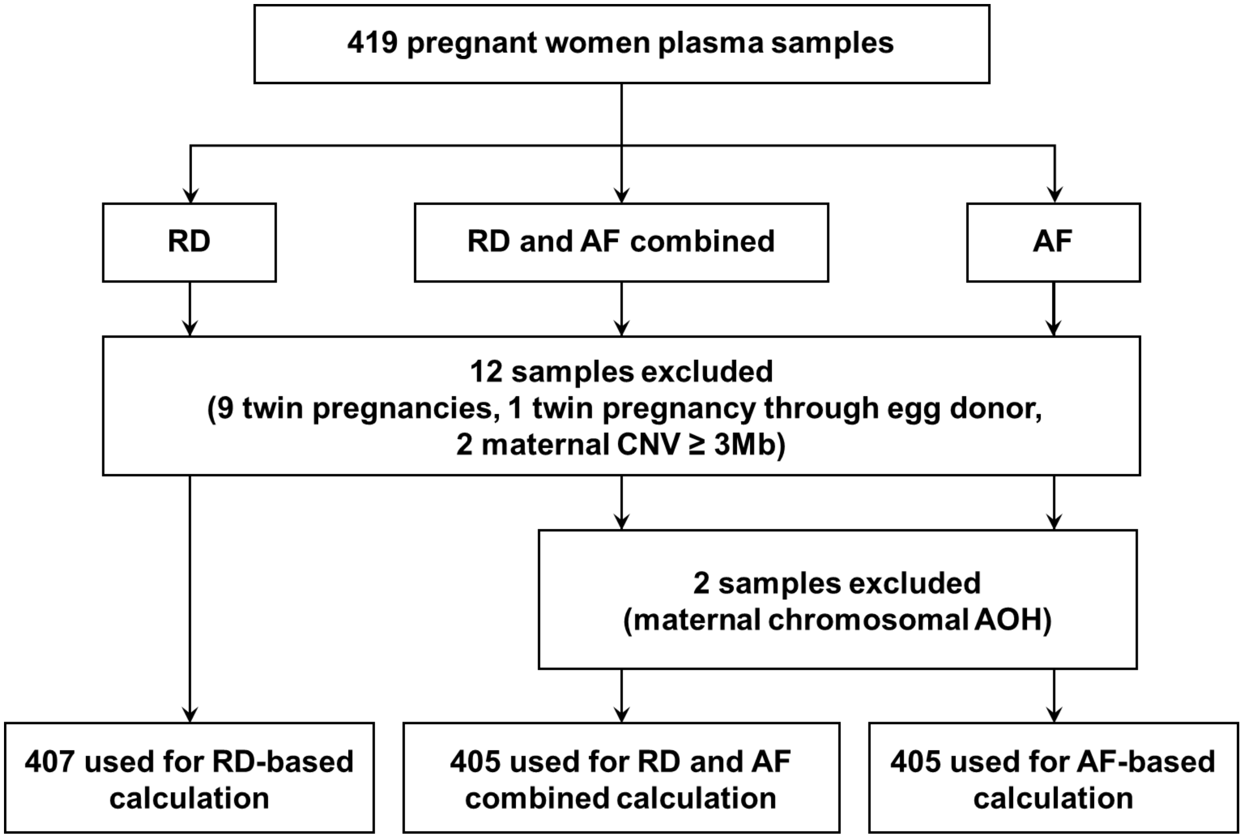

Supplementary Fig. S3 The analytical validation for chomosomal aberrations using the RD, AF, and RD and AF combined approach. A positive result was called when the RD-based method yielded a Z score above 3 or the AF-based method yielded a log likelihood value less than -15. The exclusion of dizygotic twins for the RD method analysis was based on a SNP-based quality control step checking for multiple gestations. RD: read-depth. AF: allelic fraction. CNV: copy number variation. AOH: absence of heterozygosity.

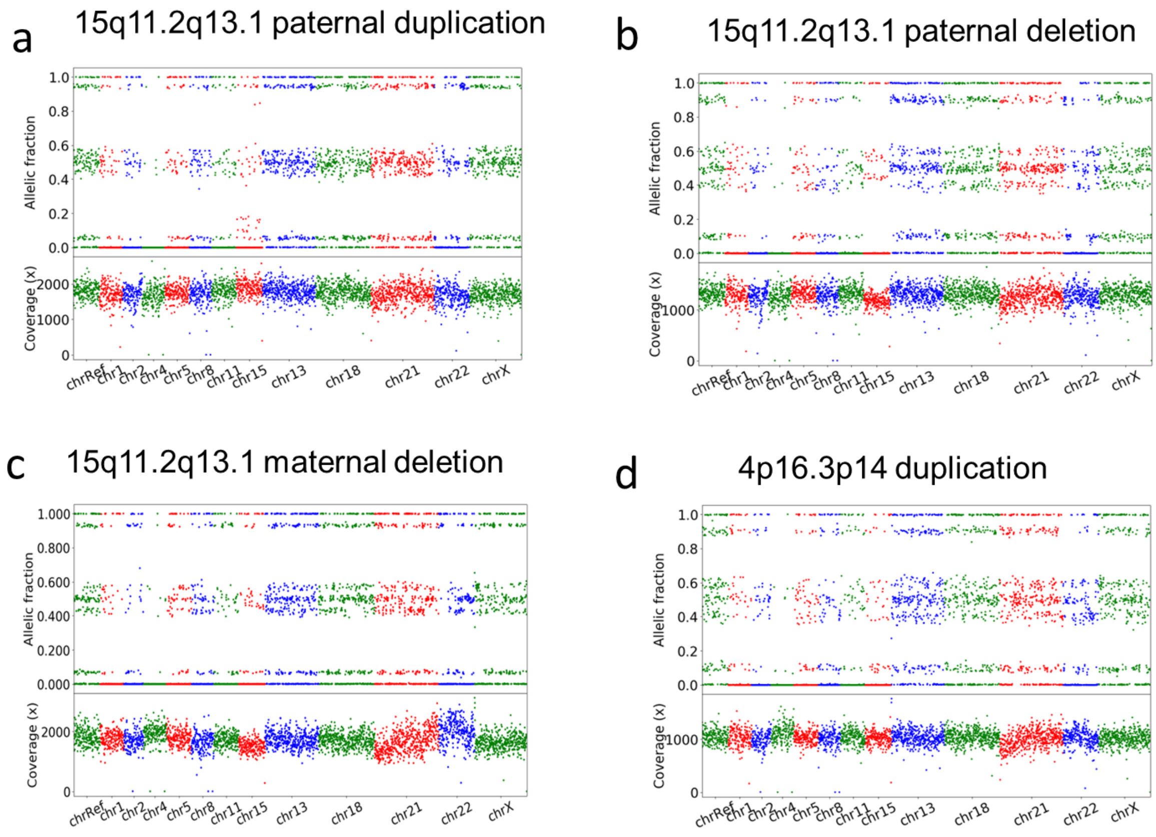

Supplementary Fig. S4 Detection of microdeletion and microduplication syndromes. a-**d** Representative cases of microdeletion and microduplication syndromes. **a** 15q11.2q12.3 duplication. **b** 15q11.2q13.1 paternal deletion (Prader-Willi syndrome). **c** 15q11.2q13.1 maternal deletion (Angelman syndrome). **d** 4p16.3p14 duplication detected with limited maternal heterozygous loci which did not affect the read-depth method. Probes were designed to target SNPs on the critical regions of chromosome 1, 2, 4, 5, 8, 13, 15, 18, 21, 22, X, and Y to screen for trisomy 13, 18, 21, sex chromosome aneuploidies, 1p36 microdeletion, 2q33.1 microdeletion, Wolf-Hirschhorn syndrome, Cri du Chat syndrome, Langer-Giedion syndrome, Jacobsen syndrome, Prader-Willi syndrome, Angelman syndrome, and DiGeorge syndrome.

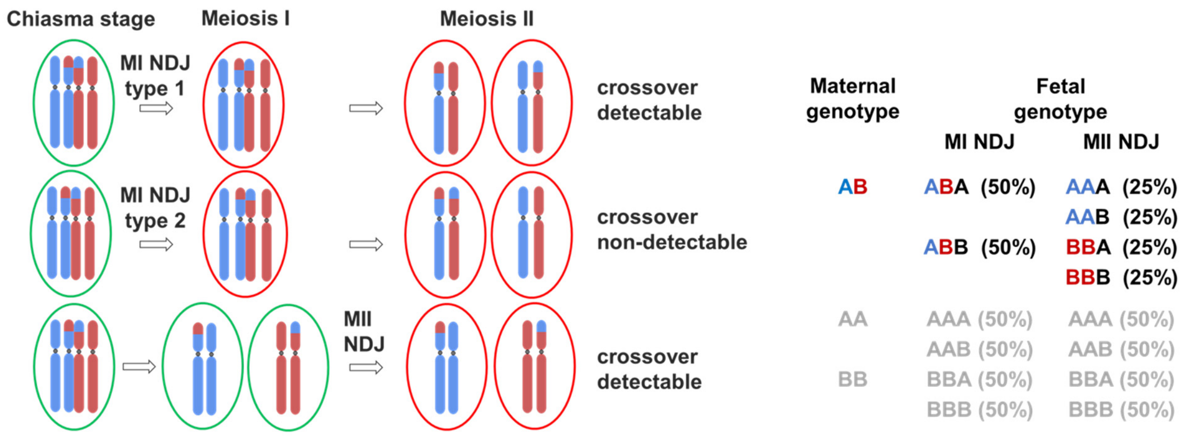

Supplementary Fig. S5 Schematic diagram of the occurrence of homologous recombination associated with maternal meiosis I (MI) or meiosis II (MII) nondisjunction (NDJ). Half of recombinants can be theoretically detected in MI NDJ (type 1) while all recombinants can be detected in MII NDJ. At the maternal heterozygous loci, the fetal genotypes with different maternal alleles (shown in red and blue letters) can be used to infer the occurrence of reombination associated with the presence of both MI and MII allelic fraction patterns.

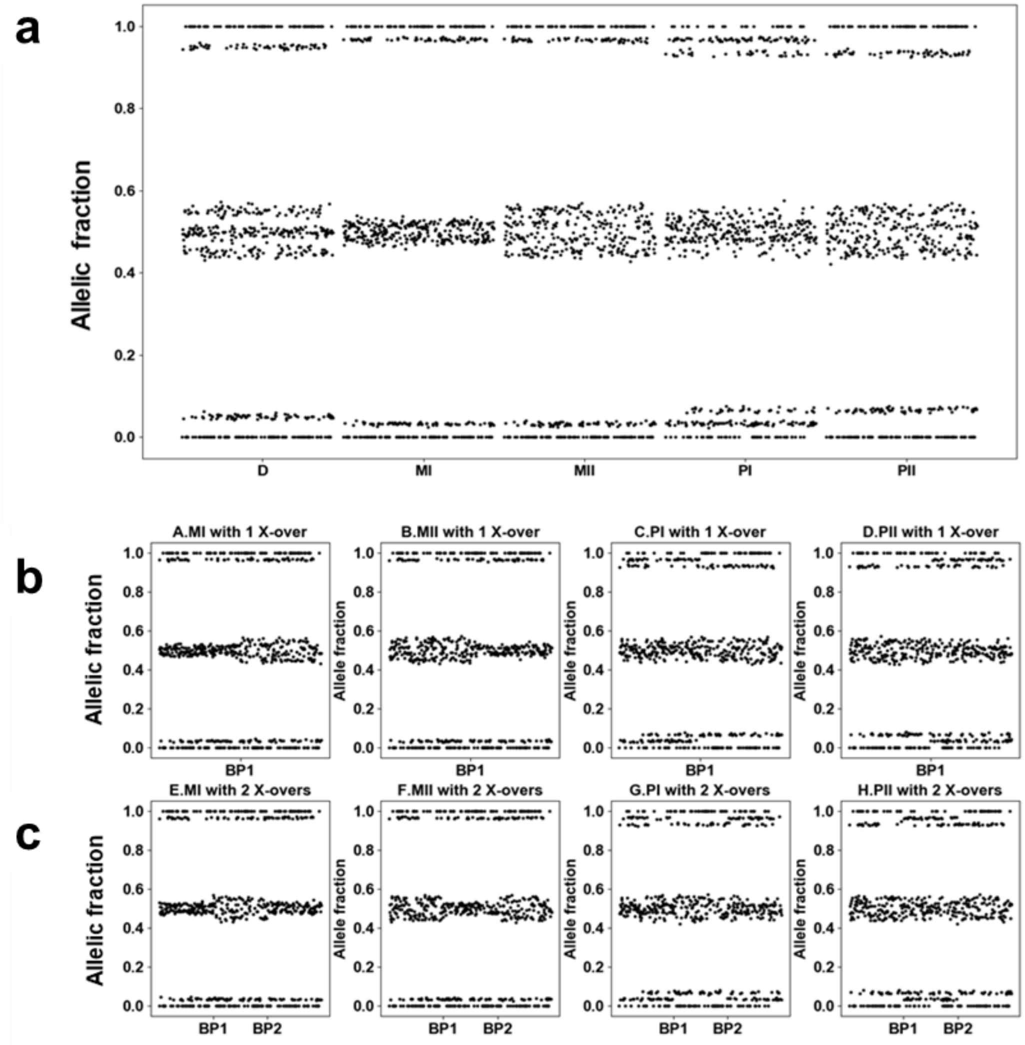

Supplementary Fig. S6 Illustration for SNP allelic fraction in aneuploidies with different meiotic errors. a The disomy and four trisomies of different parental and meiotic origins including maternal meiosis I (MI), maternal meiosis II (MII), paternal meiosis I (PI), and paternal meiosis II (PII) nondisjunction which have distinct SNP AF patterns. **b** The SNP AF patterns of a single crossover in different meiotic errors. **c** The SNP AF patterns of two crossovers in different meiotic errors. BP: break point. D: disomy. AF: allelic fraction.

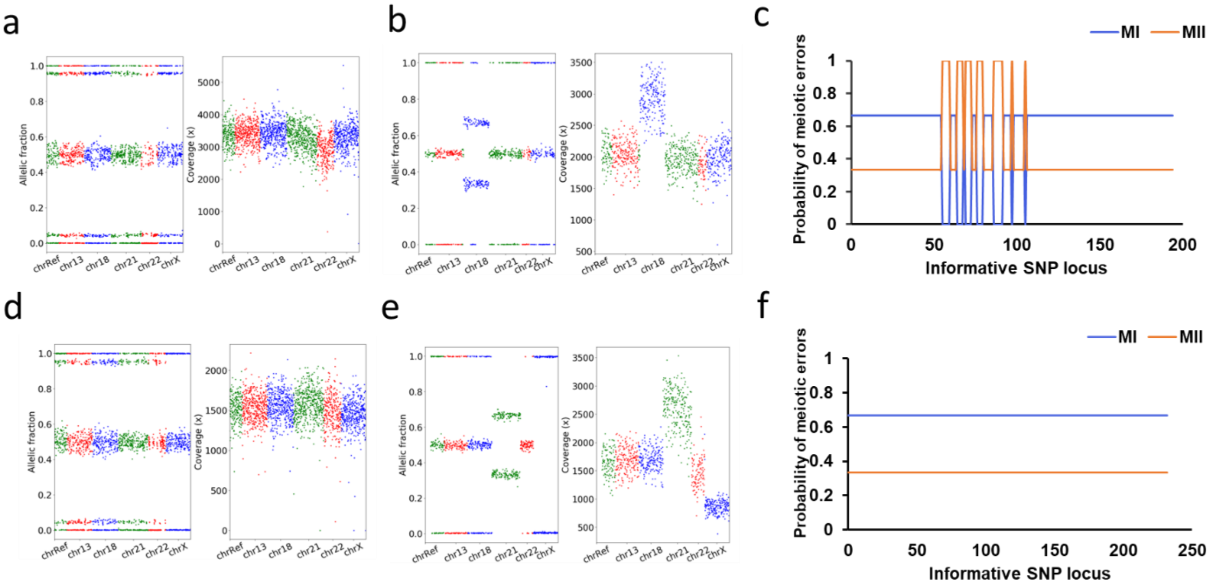

Supplementary Fig. S7 Aneuploidies with or without detected homologous recombination. a A trisomy 18 case of MII NDJ with occurrence of two crossovers on the p and q arm. **b** Confirmatory results from the respective amniocytes of the trisomy 18 case. Note that the absence of fetal homozygosity near the 18 p and q telomeric regions indicate two chromosome crossovers. **c** At loci where the mother is heterozygous, fetal homozygosity (AAA or BBB) is consistent with MII NDJ. When fetal genotype is heterozygous (ABB or AAB), the prior probability for the detectable MI NDJ is 2/3 while that for MII NDJ is 1/3 assuming an equal incidence in MI and MII NDJ. The probability of meiotic errors for each informative locus was plotted. **d**-**f** A trisomy 21 case of MI NDJ without occurrence of crossover during meiosis. Data was generated from both maternal plasma cell-free DNA (**d**) and the respective amniocytes (**e**, **f**) all of which demonstrated no occurrence of recombination. MI: maternal meiosis I. MII: maternal meiosis II. PI: paternal meiosis I. PII: paternal meiosis II. NDJ: nondisjunction.

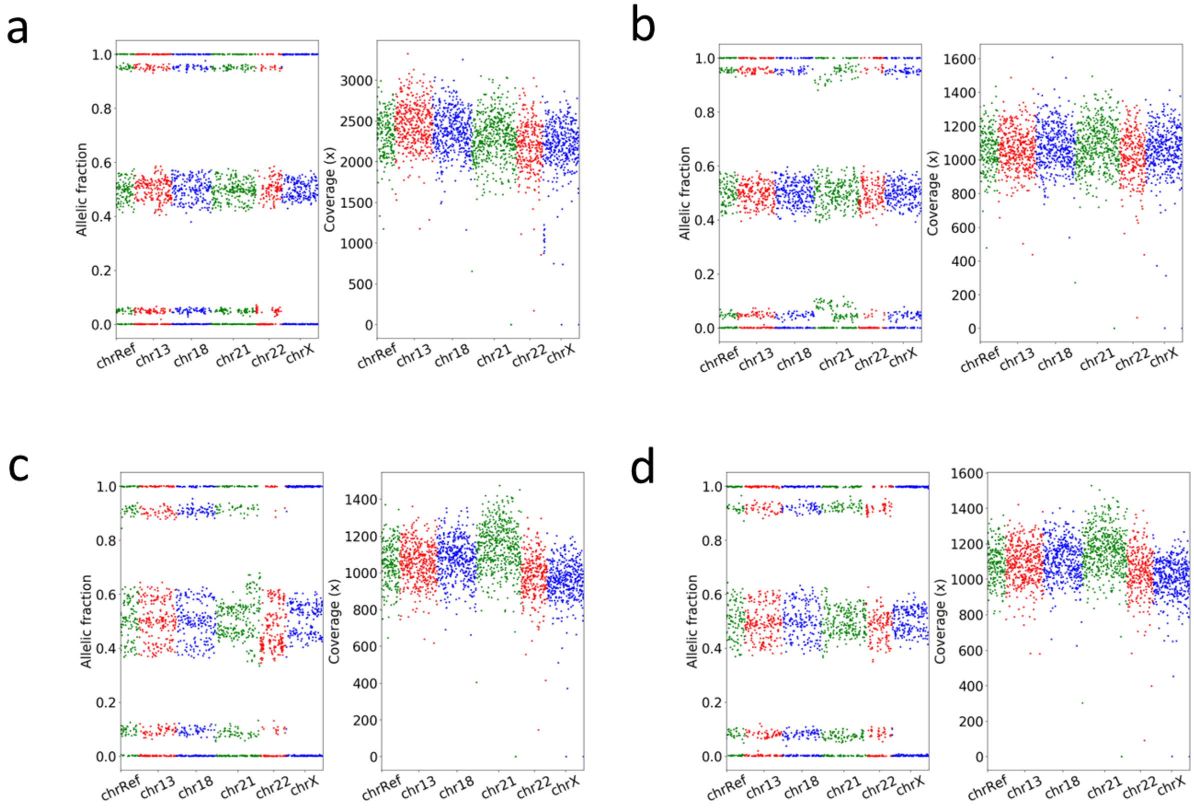

Supplementary Fig. S8 Homologous recombination detected in the plasma cell-free DNA from pregnancies with aneuploidies. a Recombination is indicated when fetal SNP AF patterns of MI NDJ and MII NDJ are both seen on chr13. **b** Recombination is indicated when fetal SNP AF patterns of PI NDJ and PII NDJ are both seen on chr21. **c** Recombination is indicated when fetal SNP AF patterns of MI NDJ and MII NDJ are both seen on chr21. **d** Recombination is indicated when fetal SNP AF patterns of MI NDJ and MII NDJ are both seen on chr21. MI: maternal meiosis I. MII: maternal meiosis II. PI: paternal meiosis I. PII: paternal meiosis II. NDJ: nondisjunction.

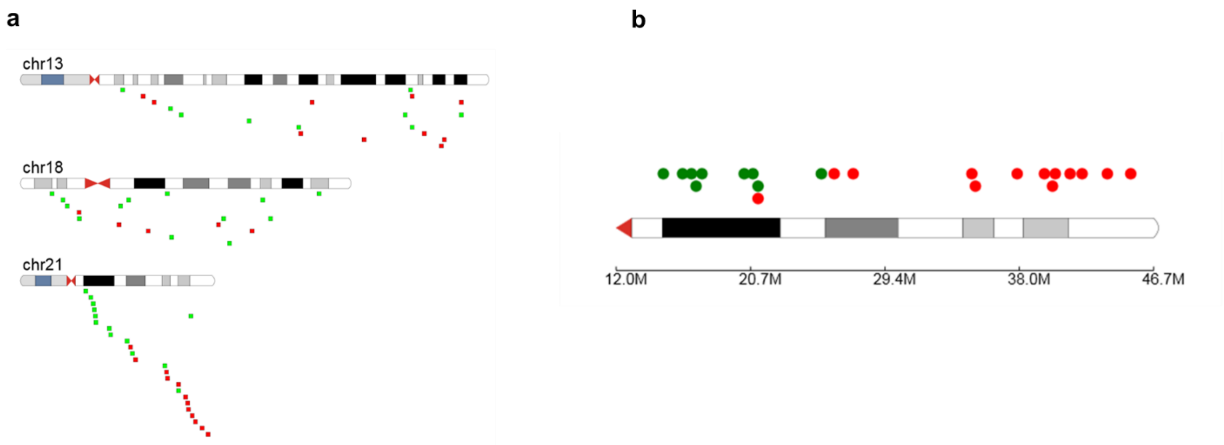

Supplementary Fig. S9 Distribution of recombination breakpoints detected in samples with common aneuploidies. a Distribution of breakpoints on chr13, chr18, and chr21 of all aneuploidy cases with detected recombinants. Red and green squares indicate the position of breakpoints found in MI and MII NDJ respectively. **b** Distribution of breakpoints on the ideogram of chr21 q arm from all trisomy 21 cases due to maternal NDJ with a single crossover detected. Red and green circles indicate the position of breakpoints for MI and MII NDJ respectively in trisomy 21 cases with a single crossover. MI: maternal meiosis I NDJ. MII: maternal meiosis II NDJ. NDJ: nondisjunction.

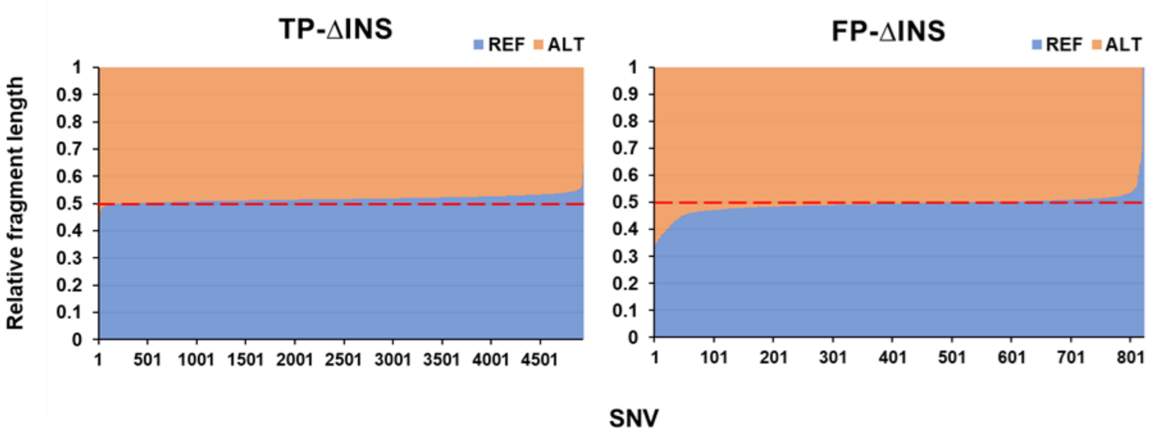

Supplementary Fig. S10 Fragments with true positive fetal variants are shorter than those with false positive variants. A total of 28 samples were used to analyze true positive and false positive variants by the median insert size of fragments harboring the reference and alternative alleles (ΔINS). To illustrate their relative fragment length, a percentage value *K* was defined as, *K* = *median insertsize of ref / (median insertsize of ref* + *median insertsize of alt)*. The X-axis represents different true positive and false positive SNV loci. The *K* and *1-K* values of the called variants were plotted in blue and yellow respectively. We observed that 95.2% of the true positive variants and 39.3% of the false positive variants exhibited a shorter fetal fragment size.TP: true positive. FP: false positive. REF: reference allele fragment. ALT: alternative allele fragment. SNV: single nucleotide variant. The dotted red line indicated the 0.5 ratio where the reference and alterative allele fragments had the same length.

Supplementary Tables

Supplementary Table S1 The characterization of meiotic errors and chromosomal recombination in aneuploidies.

	Trisomy 21 (n=47)			Trisomy 18 (n=14)			Trisomy 13 (n=12)		
Meiotic error origin		No recombination detected	Recombination detected		No recombination detected	Recombination detected		No recombination detected	Recombination detected
	Total	22 (46.8%)	25 (53.2%)	Total	5 (35.7%)	9 (64.3%)	Total	2 (16.7%)	10 (83.3%)
Maternal meiosis I	30 (63.8%)	17 (77.3%)	Cx=1, 13 (52.0%)	4 (28.6%)	1 (20.0%)	Cx=1, 1 (11.1%)<br>Cx=2, 2 (22.2%)	6 (50.0%)	1 (50.0%)	Cx=1, 1 (10.0%)<br>Cx=2, 3 (30.0%)<br>Cx=3, 1 (10.0%)
Maternal meiosis II	13 (27.7%)	3 (13.6%)	Cx=1, 9 (36.0%)<br>Cx=2, 1 (4.0%)	8 (57.1%)	2 (40.0%)	Cx=1, 2 (22.2%)<br>Cx=2, 1 (11.1%)<br>Cx=3, 3 (33.3%)	5 (41.7%)		Cx=1, 2 (10.0%)<br>Cx=2, 2 (20.0%)<br>Cx=3, 1 (10.0%)
Paternal meiosis I	1 (2.1%)	1 (4.5%)							
Paternal meiosis II	3 (6.4%)	1 (4.5%)	Cx=1, 2 (8.0%)	2 (14.2%)	2 (40.0%)		1 (8.3%)	1 (50.0%)	

Cx: the number of crossovers detected.

Supplementary Table S2 The validation for the detection of monogenic variants.

Parameter	Calculation	Results
True positive variants		5,157
True negative variants		4,0321,704
False positive variants without filtering (FP_NoFLT)		823
False positive variants with ACD & FMID filtering (FP_FullFLT)		526
False negative variants		25
Analytical sensitivity	TP/(TP+FN)	99.5% (95%CI, 99.3%-99.7%)
Analytical specificity	TN/(TN+FP_FullFLT)	99.9% (95%CI, 99.9%-100%)
Analytical positive predictive value without filtering	TP/(TP+FP_NoFLT)	86.2% (95%CI, 85.3%-87.1%)
Analytical positive predictive value with ACD & FMID filtering	TP/(TP+FP_FullFLT)	90.7% (95%CI, 90.0%-91.5%)
Analytical negative predictive value	TN/(TN+FN)	>99.9% (95%CI, 99.9%-100%)

ACD: allele count distribution. FMID: Fetal-Maternal Insert-size Distribution. FP: false positive. FN: false negative. TP: true positive. FP: false positive. NoFLT: no variant filter applied. FullFLT: all variant filters applied. See Methods for details.

Supplementary Table S3 Allele count distribution and insert-size filtering results.

Filtering method	Fetal variants filtered out	Non-fetal variants filtered out
FMID-ΔIns	303/5,182	536/823
FMID-MinP	1648/5,182	699/823
FMID	286/5,182	475/823
ACD	143/5,182	503/823
ACD & FMID	4/5,182	297/823

ACD: allele count distribution. FMID: fetal-maternal insert-size distribution. ΔIns: insert-size comparison to check if alternative allele fragments are shorter than the reference allele fragments. MinP: insert-size comparison to check if alternative allele fragments are significantly different from the reference allele fragments in length. See Methods for details.

Supplementary Table S4 Intra- and inter-assay reproducibility validation.

Sample ID	S1	S1	S1	S1	S1
Run ID	R1	R1	R1	R2	R3
Performed by	T1	T1	T1	T2	T2
Mean depth	2,517	2,436	2,580	3,496	2,535
Fetal fraction (%)	10.7	10.7	10.7	10.6	10.9
Abnormality detected	Trisomy 21	Trisomy 21	Trisomy 21	Trisomy 21	Trisomy 21
Pathogenic variant frequency (%)	N/A	N/A	N/A	N/A	N/A

Sample ID	S2	S2	S2	S2	S2
Run ID	R1	R1	R1	R2	R3
Performed by	T1	T1	T1	T2	T2
Mean depth	2,244	2,227	2,313	2,925	2,400
Fetal fraction (%)	6.9	7.1	6.9	7.1	6.8
Abnormality detected	Trisomy 18	Trisomy 18	Trisomy 18	Trisomy 18	Trisomy 18
Pathogenic variant frequency (%)	N/A	N/A	N/A	N/A	N/A

Sample ID	S3	S3	S3	S3	S3
Run ID	R1	R2	R2	R2	R3
Performed by	T1	T1	T1	T1	T2
Mean depth	2,297	3,351	2,966	1,920	2,901
Fetal fraction (%)	9.1	9.3	9.1	9.5	9.1
Abnormality detected	Trisomy 13	Trisomy 13	Trisomy 13	Trisomy 13	Trisomy 13
Pathogenic variant frequency (%)	N/A	N/A	N/A	N/A	N/A

Sample ID	S4	S4	S4	S4	S4
Run ID	R1	R2	R3	R3	R3
Performed by	T1	T1	T2	T2	T2
Mean depth	2,533	3,147	2,823	2,283	2,465
Fetal fraction (%)	10.3	10.5	10.4	10.5	10.3
Abnormality detected	22q11.2del	22q11.2del	22q11.2del	22q11.2del	22q11.2del
Pathogenic variant frequency (%)	N/A	N/A	N/A	N/A	N/A

Sample ID	S5	S5	S5	S5	S5
Run ID	R1	R1	R1	R2	R3
Performed by	T1	T1	T1	T2	T2
Mean depth	1,718	3,851	2,625	3,747	3,547
Fetal fraction (%)	9.4	9.4	9.3	9.4	9.3
Abnormality detected	*FGFR3*: c.1138G>A	*FGFR3*: c.1138G>A	*FGFR3*: c.1138G>A	*FGFR3*: c.1138G>A	*FGFR3*: c.1138G>A
Pathogenic variant frequency (%)	4.7	4.8	4.0	4.7	4.7

Supplementary Table S5 Demographic and clinical characteristics of the subjects.

Characteristic	Value
Number of subjects analyzed	1,129
Mean maternal age - year (range)	32.5 (17-47)
Mothers ≥35 years old - no. (%)	426 (37.7)
Mean gestational age at sample collection (range) - week	15.3 (11.1-32.7)
Pregnancies with abnormal ultrasound screening results - no. (%)	267 (23.6)
Pregnancies with positive NIPS screening results – no. (%)	30 (2.7)
Pregnancies with positive family history - no. (%)	1 (0.09)

Supplementary Table S6 Clinical performance of the NIPS test in 1,129 pregnancies.

Parameter	Calculation	Results
True positive cases (TP)		70
True negative cases (TN)		1,052
False positive cases (FP)		7
False negative cases (FN)		0
Sensitivity (n=70)	TP / (TP + FN)	100% (94.9-100%)
Specificity (n=1,052)	TN / (TN + FP)	99.3% (98.6-99.7%)

Supplementary Table S7 The comparison of the NIPS in this work and previous NIPS methods.

	Current method	PMID: 19073917 [1], 24843150 [2], 30593563 [3], 29760053 [4]	PMID: 24805989 [5]	PMID: 22674554 [6]	PMID: 32024963 [7]	PMID: 30787035 [8]	PMID: 31832098 [9]	PMID: 31283802 [10]
**Technical innovation**	Innovative target enrichment suppressing allelic hybridization bias for highly accurate cfDNA SNP quantification	Development of WGS method for fetal cfDNA genetic analyses	Development of multiplexed PCR based NGS method for aneuploidy detection using cfDNA	N/A	N/A	N/A	N/A	N/A
**Analytical innovation**	New fetal cfDNA analytical algorithms integrating SNP allelic fraction, read-depth, SNP linkage, and fragment analyses to deconvolute fetal genome from maternal background and detect fetal CNV and SNV	Development of read-depth and SNP- based copy number analysis for aneuploidy and cfDNA fragment analysis	Development of SNP based algorithm for the detection of fetal aneuploidies and CNVs	Using the haplotype-resolved maternal genome sequencing, shotgun paternal genome sequencing, and deep sequencing of maternal plasma to infer fetal genome	Fetal cfDNA haplotyping analysis using maternal plasma DNA along with genomic DNA from family members	Bayesian method for the NIPD of monogenic diseases that is independent of the mode of inheritance and parental origin	Multi-engine bioinformatics analysis pipeline for chromosomal aneuploidies, microdeletions, and autosomal recessive disorders	Development of a computational model which incorporates sequential read counts and allelic ratios as inputs to call aneuploidies
**Scientific discovery**	Discovery and characterization of homologous recombination associated with meiotic chromosome nondisjunction in fetal cfDNA	Discovery of cfDNA in pregnant women's peripheral blood and fetal cfDNA fragmentation pattern	Discovery of meiotic non-disjunction in the cfDNA analysis	N/A	N/A	N/A	N/A	N/A
**Clinical significance**	Clinical validation with 1,129 cases which demonstrated high test accuracy for the concurrent detection of aneuploidy, microdeletion, and monogenic disorders	First utilizing fetal cfDNA for human genetic screening and cancer early detection	Development and implementation of SNP-based NIPS for aneuploidies	Proof of concept for noninvasive whole genome analysis for fetal genetic disease	Proof of concept for fetal haplotyping and copy-number profiling of the fetal genome	Proof of concept for noninvasive analyses of fetal monogenic diseases	Proof of concept for a comprehensive NIPS with analytical validation	Proof of concept for the use of high-coverage sequencing data to call aneuploidies using simulation assays

Supplementary Table S8 The expected allelic fraction of fetal SNPs in cell-free DNA.

Maternal genotype	Fetal ploidy	Fetal genotype								
		AA	AB	BB	AAA	AAB	ABB	BBB	A	B
AA	D	1	1-FF/2	-	-	-	-	-	-	-
AB	D	c+FF/2	c	c-FF/2	-	-	-	-	-	-
BB	D	-	FF/2	0	-	-	-	-	-	-
AA	MI	-	-	-	1	1-FF_c/3	-	-	-	-
AB	MI	-	-	-	-	c+FF_c/6	c-FF_c/6	-	-	-
BB	MI	-	-	-	-	-	FF_c/3	0	-	-
AA	MII	-	-	-	1	1-FF_c/3	-	-	-	-
AB	MII	-	-	-	c+FF_c/2	c+FF_c/6	c-FF_c/6	c-FF_c/2	-	-
BB	MII	-	-	-	-	-	FF_c/3	0	-	-
AA	PI	-	-	-	1	1-FFc/3	1-2FF_c/3	-	-	-
AB	PI	-	-	-	c+FF_c/2	c+FFc/6	c-FF_c/6	c-FF_c/2	-	-
BB	PI	-	-	-	-	2ffc/3	FF_c/3	0	-	-
AA	PII	-	-	-	1	-	1-2FF_c/3	-	-	-
AB	PII	-	-	-	c+FF_c/2	c+FF_c/6	c-FF_c/6	c-FF_c/2	-	-
BB	PII	-	-	-	-	2FF_c/3	-	0	-	-
AA	LM	-	-	-	-	-	-	-	1	1-FF_c
AB	LM	-	-	-	-	-	-	-	c+FF_c/2	c-FF_c/2
BB	LM	-	-	-	-	-	-	-	FF_c	0
AA	LP	-	-	-	-	-	-	-	1	0
AB	LP	-	-	-	-	-	-	-	c+FF_c/2	c-FF_c/2
BB	LP	-	-	-	-	-	-	-	-	-

D: disomy. MI: maternal meiosis I NDJ. MII: maternal meiosis II NDJ. PI: paternal meiosis I NDJ. PII: paternal meiosis II NDJ. LM: loss of a maternal chromosome. LP: loss of a paternal chromosome. NDJ: nondisjunction. FF: fetal fraction. FF_c: corrected fetal fraction when fetus has an aneuploidy (see Methods). C: the central allelic fraction. For a series of samples within the same hybridization pool which all show maternal heterozygosity for a SNP, then the median of allelic fractions of this SNP in these samples is used as the central allelic fraction. For SNPs on a trisomy or monosomy chromosome, the corrected FF (denoted by FFc) is computed for trisomy, FFc=3FF/(2+FF) and monosomy, FFc=FF/(2-FF).

Supplementary Table S9 Fetal genotype probability in different ploidy states.

Maternal genotype	Paternal genotype	Fetal ploidy	Fetal genotype								
			AA	AB	BB	AAA	AAB	ABB	BBB	A	B
AA	AA	D	1	0	0	0	0	0	0	0	0
AA	AB	D	0.5	0.5	0	0	0	0	0	0	0
AA	BB	D	0	1	0	0	0	0	0	0	0
AB	AA	D	0.5	0.5	0	0	0	0	0	0	0
AB	AB	D	0.25	0.5	0.25	0	0	0	0	0	0
AB	BB	D	0	0.5	0.5	0	0	0	0	0	0
BB	AA	D	0	1	0	0	0	0	0	0	0
BB	AB	D	0	0.5	0.5	0	0	0	0	0	0
BB	BB	D	0	0	1	0	0	0	0	0	0
AA	AA	MI	0	0	0	1	0	0	0	0	0
AA	AB	MI	0	0	0	0.5	0.5	0	0	0	0
AA	BB	MI	0	0	0	0	1	0	0	0	0
AB	AA	MI	0	0	0	0	1	0	0	0	0
AB	AB	MI	0	0	0	0	0.5	0.5	0	0	0
AB	BB	MI	0	0	0	0	0	1	0	0	0
BB	AA	MI	0	0	0	0	0	1	0	0	0
BB	AB	MI	0	0	0	0	0	0.5	0.5	0	0
BB	BB	MI	0	0	0	0	0	0	1	0	0
AA	AA	MII	0	0	0	1	0	0	0	0	0
AA	AB	MII	0	0	0	0.5	0.5	0	0	0	0
AA	BB	MII	0	0	0	0	1	0	0	0	0
AB	AA	MII	0	0	0	0.5	0	0.5	0	0	0
AB	AB	MII	0	0	0	0.25	0.25	0.25	0.25	0	0
AB	BB	MII	0	0	0	0	0.5	0	0.5	0	0
BB	AA	MII	0	0	0	0	0	1	0	0	0
BB	AB	MII	0	0	0	0	0	0.5	0.5	0	0
BB	BB	MII	0	0	0	0	0	0	1	0	0
AA	AA	PI	0	0	0	1	0	0	0	0	0
AA	AB	PI	0	0	0	0	1	0	0	0	0
AA	BB	PI	0	0	0	0	0	1	0	0	0
AB	AA	PI	0	0	0	0.5	0.5	0	0	0	0
AB	AB	PI	0	0	0	0	0.5	0.5	0	0	0
AB	BB	PI	0	0	0	0	0	0.5	0.5	0	0
BB	AA	PI	0	0	0	0	1	0	0	0	0
BB	AB	PI	0	0	0	0	0	1	0	0	0
BB	BB	PI	0	0	0	0	0	0	1	0	0
AA	AA	PII	0	0	0	1	0	0	0	0	0
AA	AB	PII	0	0	0	0.5	0	0.5	0	0	0
AA	BB	PII	0	0	0	0	0	1	0	0	0
AB	AA	PII	0	0	0	0.5	0.5	0	0	0	0
AB	AB	PII	0	0	0	0.25	0.25	0.25	0.25	0	0
AB	BB	PII	0	0	0	0	0	0.5	0.5	0	0
BB	AA	PII	0	0	0	0	1	0	0	0	0
BB	AB	PII	0	0	0	0	0.5	0	0.5	0	0
BB	BB	PII	0	0	0	0	0	0	1	0	0
AA	AA	LM	0	0	0	0	0	0	0	1	0
AA	AB	LM	0	0	0	0	0	0	0	0.5	0.5
AA	BB	LM	0	0	0	0	0	0	0	0	1
AB	AA	LM	0	0	0	0	0	0	0	1	0
AB	AB	LM	0	0	0	0	0	0	0	0.5	0.5
AB	BB	LM	0	0	0	0	0	0	0	0	1
BB	AA	LM	0	0	0	0	0	0	0	1	0
BB	AB	LM	0	0	0	0	0	0	0	0.5	0.5
BB	BB	LM	0	0	0	0	0	0	0	0	1
AA	AA	LP	0	0	0	0	0	0	0	1	0
AA	AB	LP	0	0	0	0	0	0	0	1	0
AA	BB	LP	0	0	0	0	0	0	0	1	0
AB	AA	LP	0	0	0	0	0	0	0	0.5	0.5
AB	AB	LP	0	0	0	0	0	0	0	0.5	0.5
AB	BB	LP	0	0	0	0	0	0	0	0.5	0.5
BB	AA	LP	0	0	0	0	0	0	0	0	1
BB	AB	LP	0	0	0	0	0	0	0	0	1
BB	BB	LP	0	0	0	0	0	0	0	0	1

D: disomy. MI: maternal meiosis I NDJ. MII: maternal meiosis II NDJ. PI: paternal meiosis I NDJ. PII: paternal meiosis II NDJ. LM: loss of a maternal chromosome. LP: loss of a paternal chromosome. NDJ: nondisjunction.

References

1 Chiu, R. W. *et al.* Noninvasive prenatal diagnosis of fetal chromosomal aneuploidy by massively parallel genomic sequencing of DNA in maternal plasma. *Proc Natl Acad Sci U S A* **105**, 20458-20463, doi:10.1073/pnas.0810641105 (2008).

2 Yu, S. C. *et al.* Size-based molecular diagnostics using plasma DNA for noninvasive prenatal testing. *Proc Natl Acad Sci U S A* **111**, 8583-8588, doi:10.1073/pnas.1406103111 (2014).

3 Serpas, L. *et al.* Dnase1l3 deletion causes aberrations in length and end-motif frequencies in plasma DNA. *Proc Natl Acad Sci U S A* **116**, 641-649, doi:10.1073/pnas.1815031116 (2019).

4 Sun, K. *et al.* Size-tagged preferred ends in maternal plasma DNA shed light on the production mechanism and show utility in noninvasive prenatal testing. *Proc Natl Acad Sci U S A* **115**, E5106-E5114, doi:10.1073/pnas.1804134115 (2018).

5 Hall, M. P. *et al.* Non-invasive prenatal detection of trisomy 13 using a single nucleotide polymorphism- and informatics-based approach. *PLoS One* **9**, e96677, doi:10.1371/journal.pone.0096677 (2014).

6 Kitzman, J. O. *et al.* Noninvasive whole-genome sequencing of a human fetus. *Sci Transl Med* **4**, 137ra176, doi:10.1126/scitranslmed.3004323 (2012).

7 Che, H. *et al.* Noninvasive prenatal diagnosis by genome-wide haplotyping of cell-free plasma DNA. *Genet Med* **22**, 962-973, doi:10.1038/s41436-019-0748-y (2020).

8 Rabinowitz, T. *et al.* Bayesian-based noninvasive prenatal diagnosis of single-gene disorders. *Genome Res* **29**, 428-438, doi:10.1101/gr.235796.118 (2019).

9 Koumbaris, G. *et al.* Targeted capture enrichment followed by NGS: development and validation of a single comprehensive NIPT for chromosomal aneuploidies, microdeletion syndromes and monogenic diseases. *Mol Cytogenet* **12**, 48, doi:10.1186/s13039-019-0459-8 (2019).

10 Teder, H. *et al.* Computational framework for targeted high-coverage sequencing based NIPT. *PLoS One* **14**, e0209139, doi:10.1371/journal.pone.0209139 (2019).
